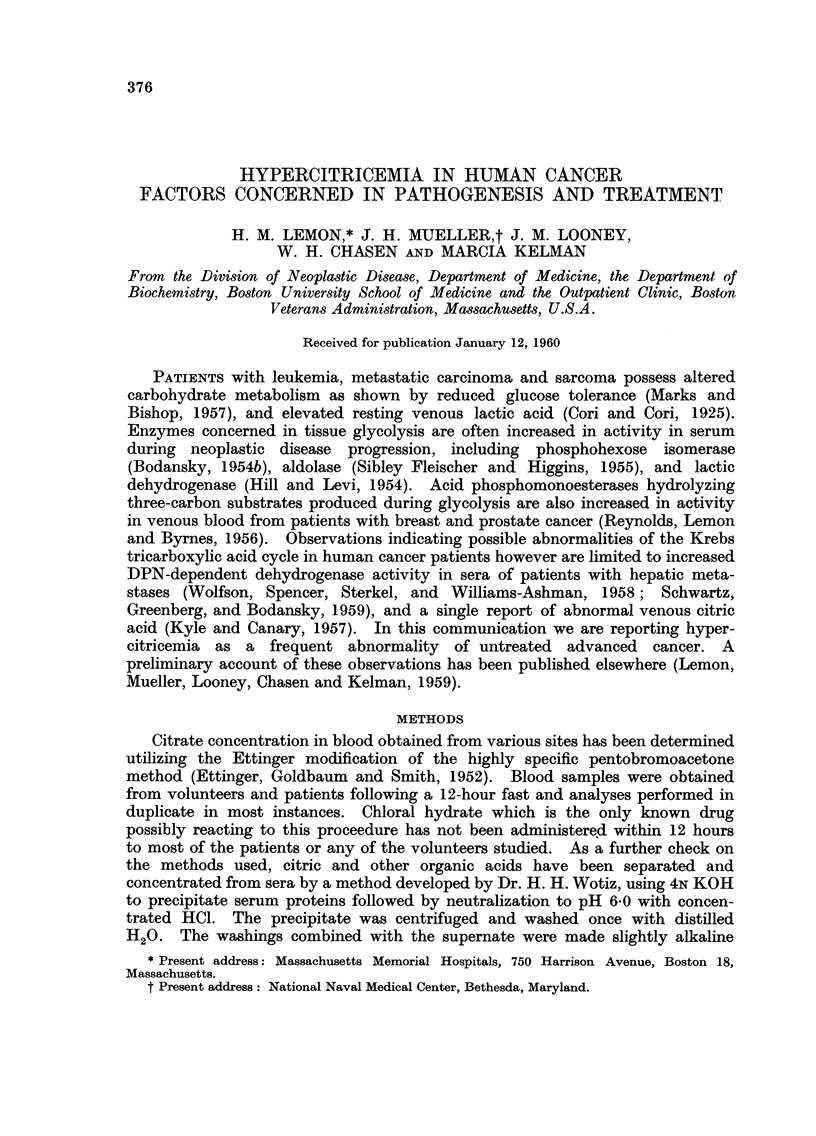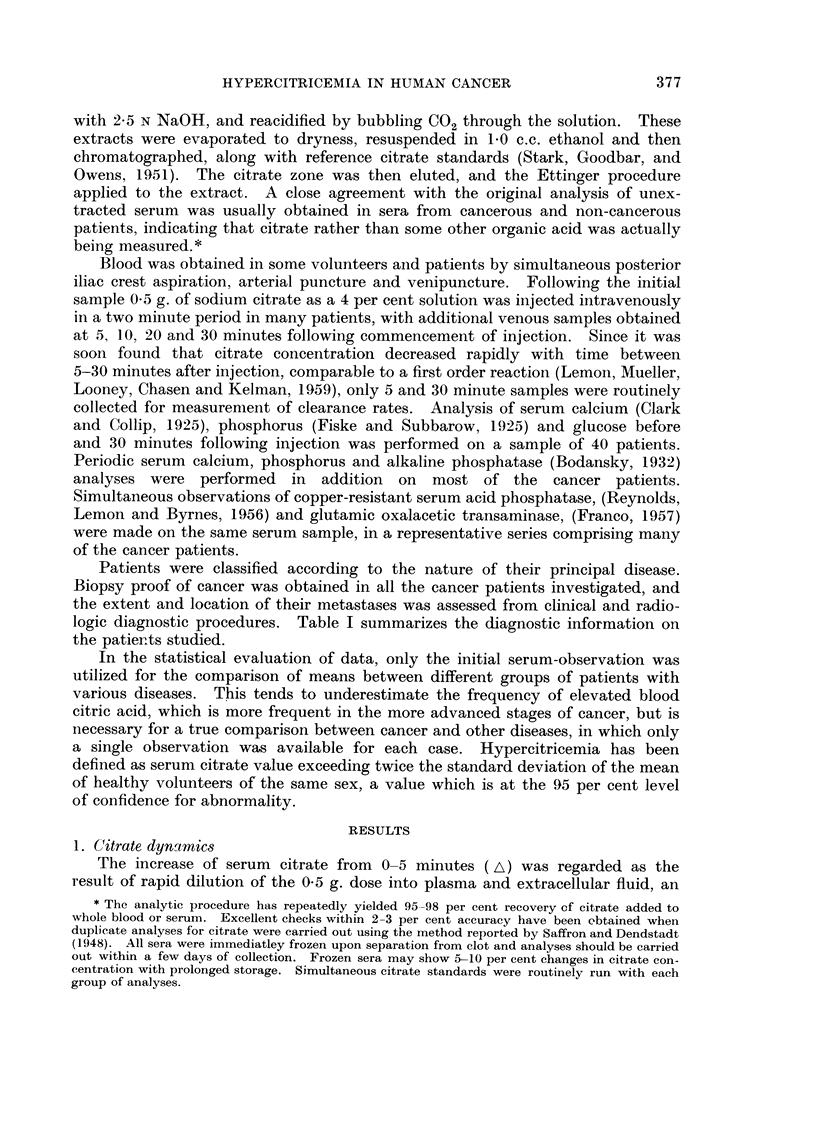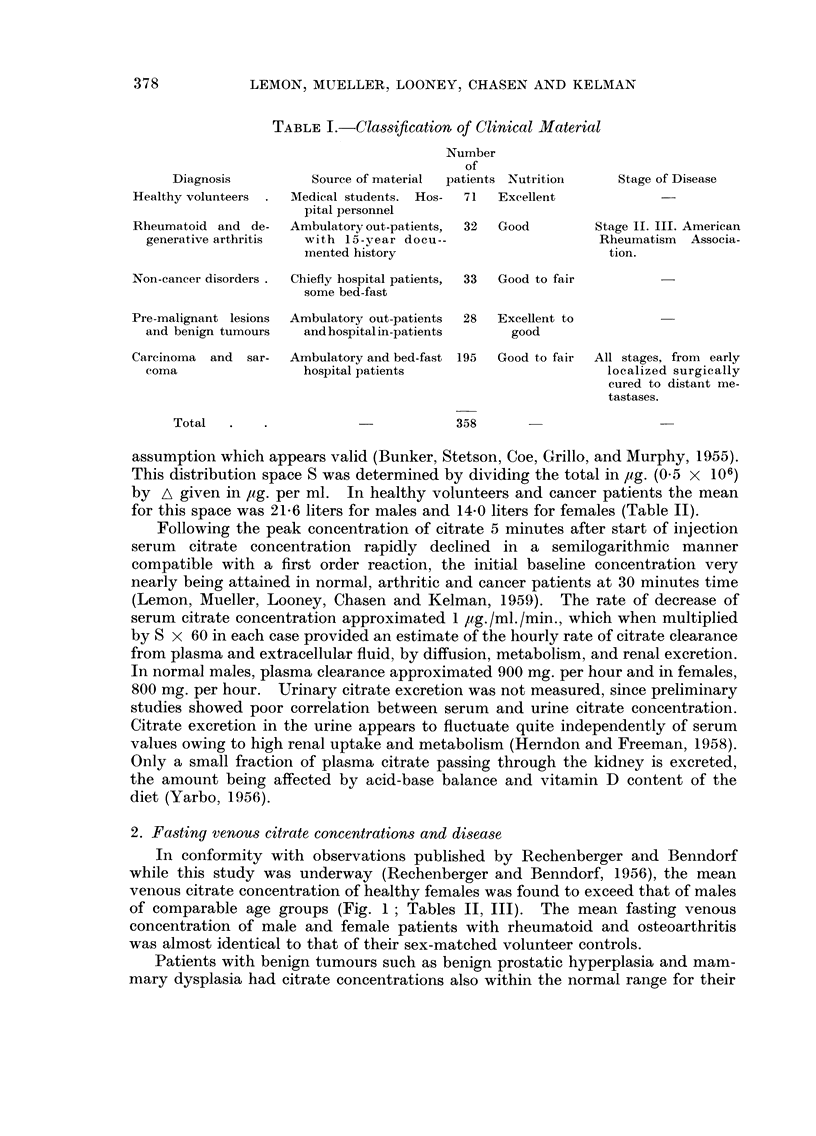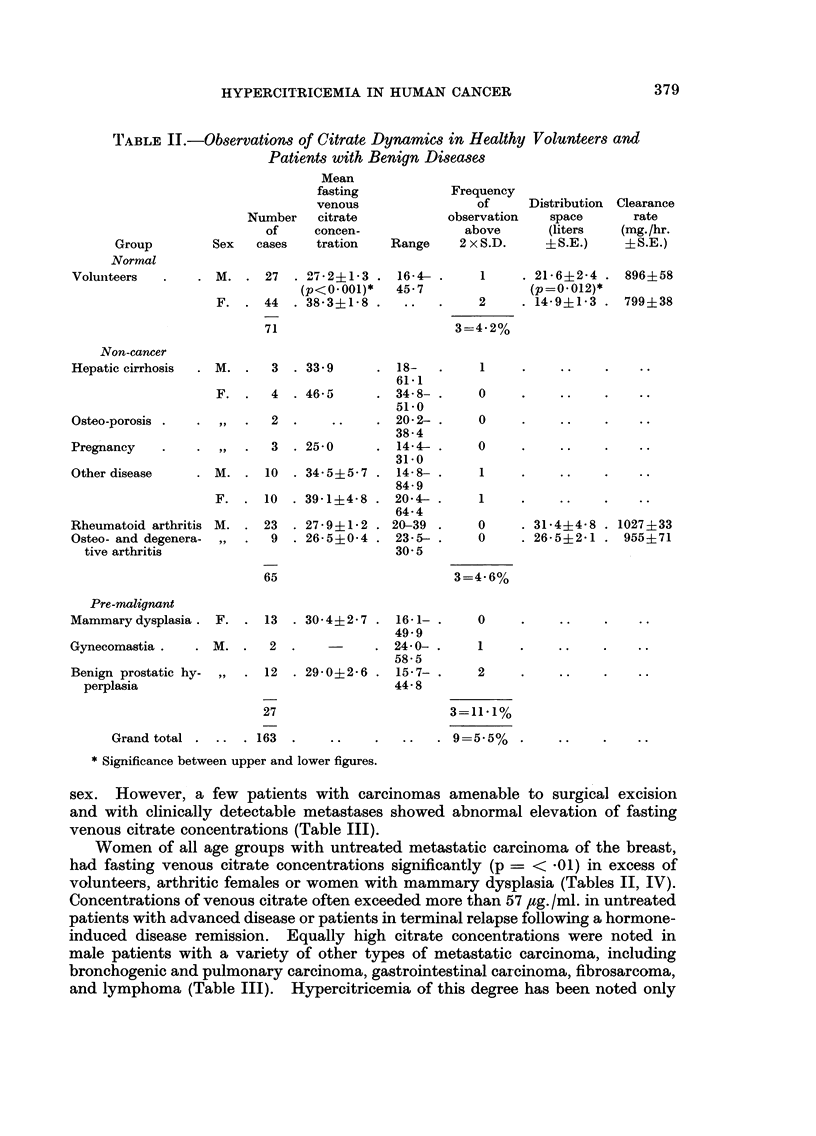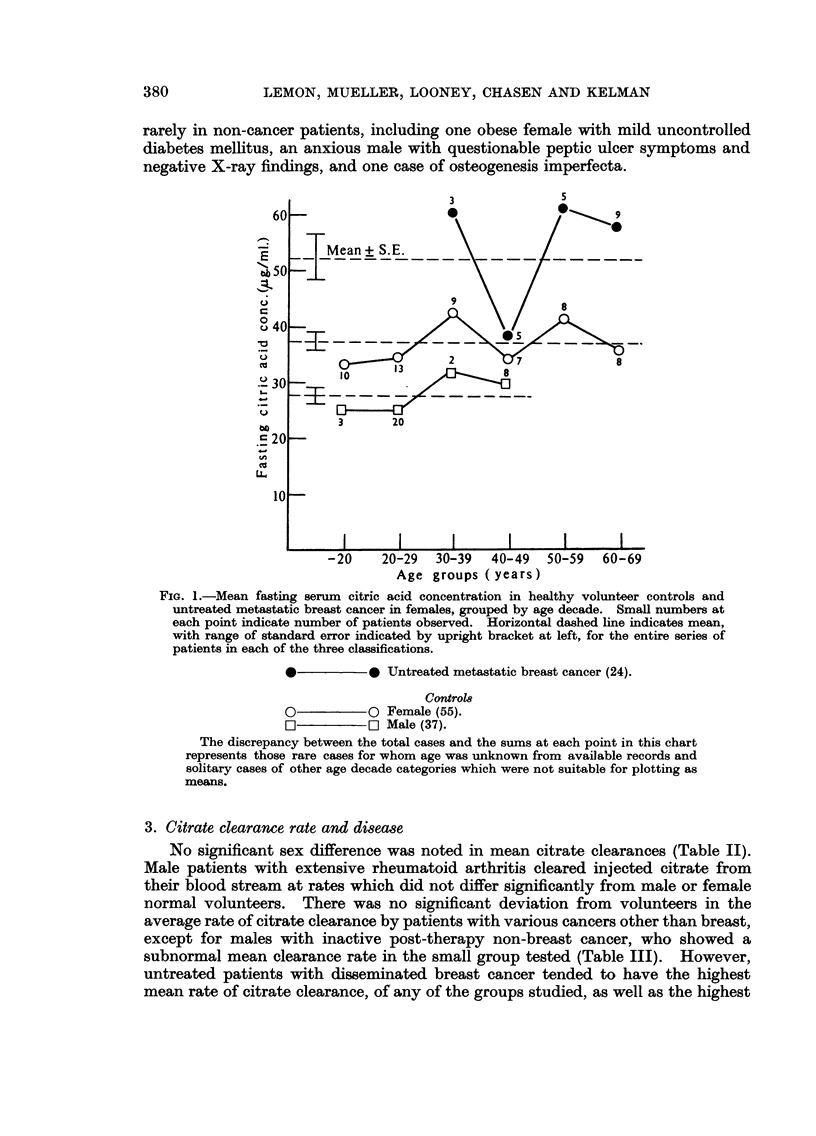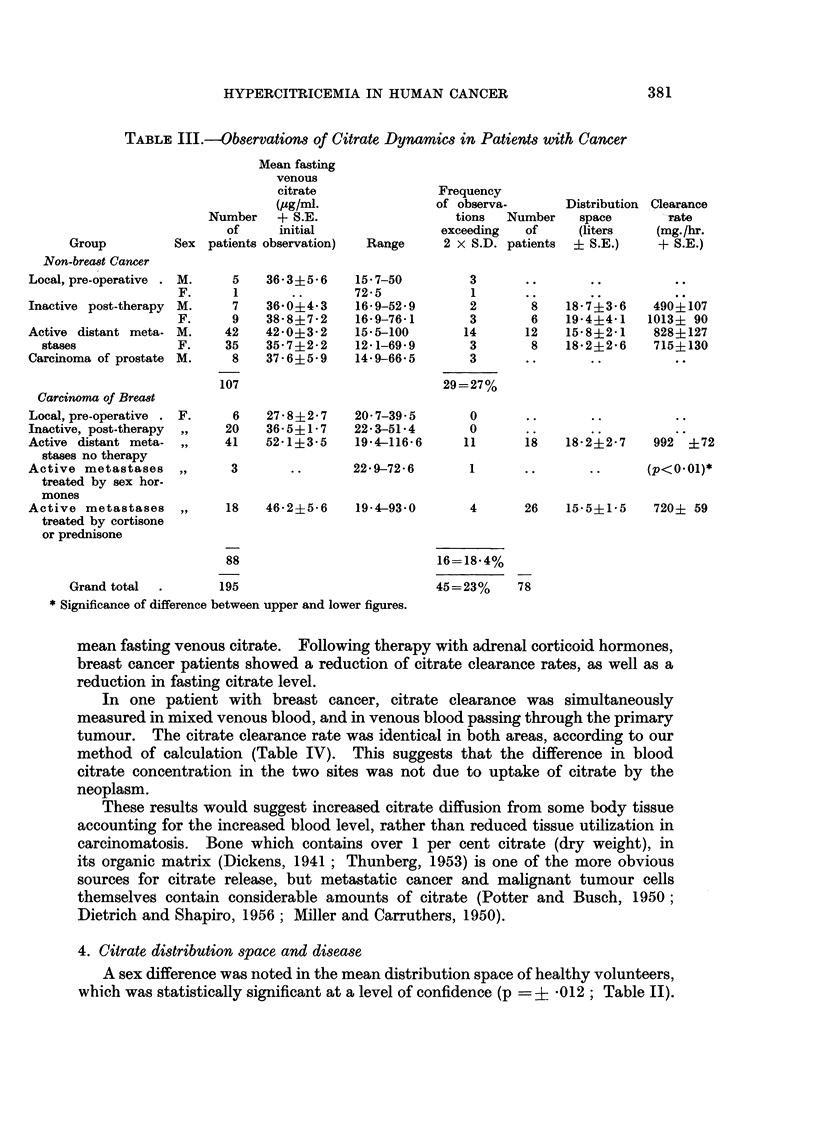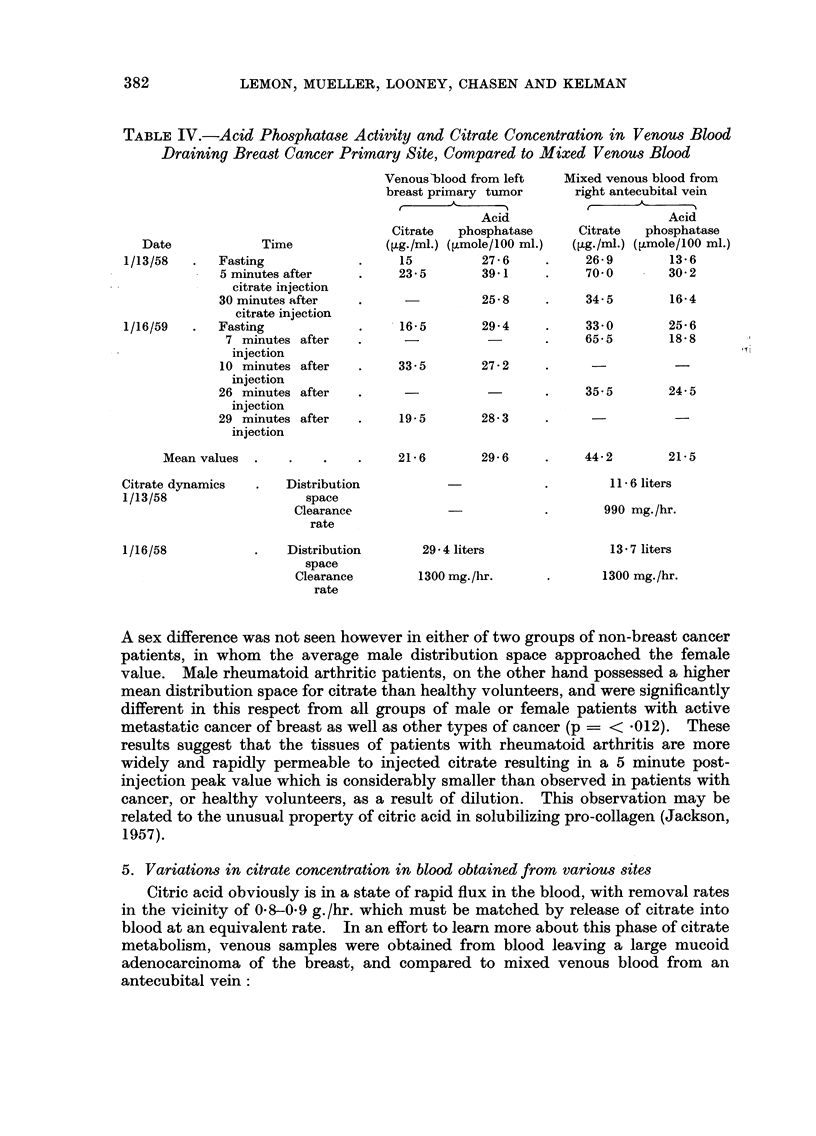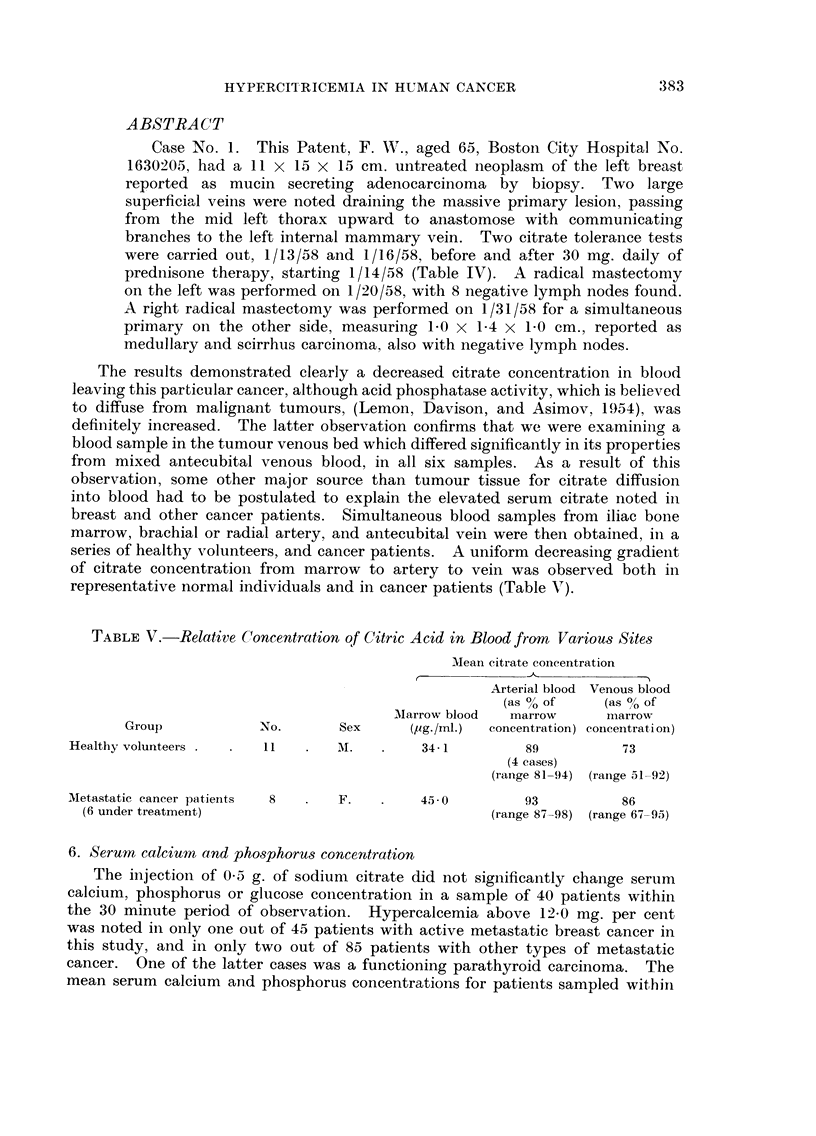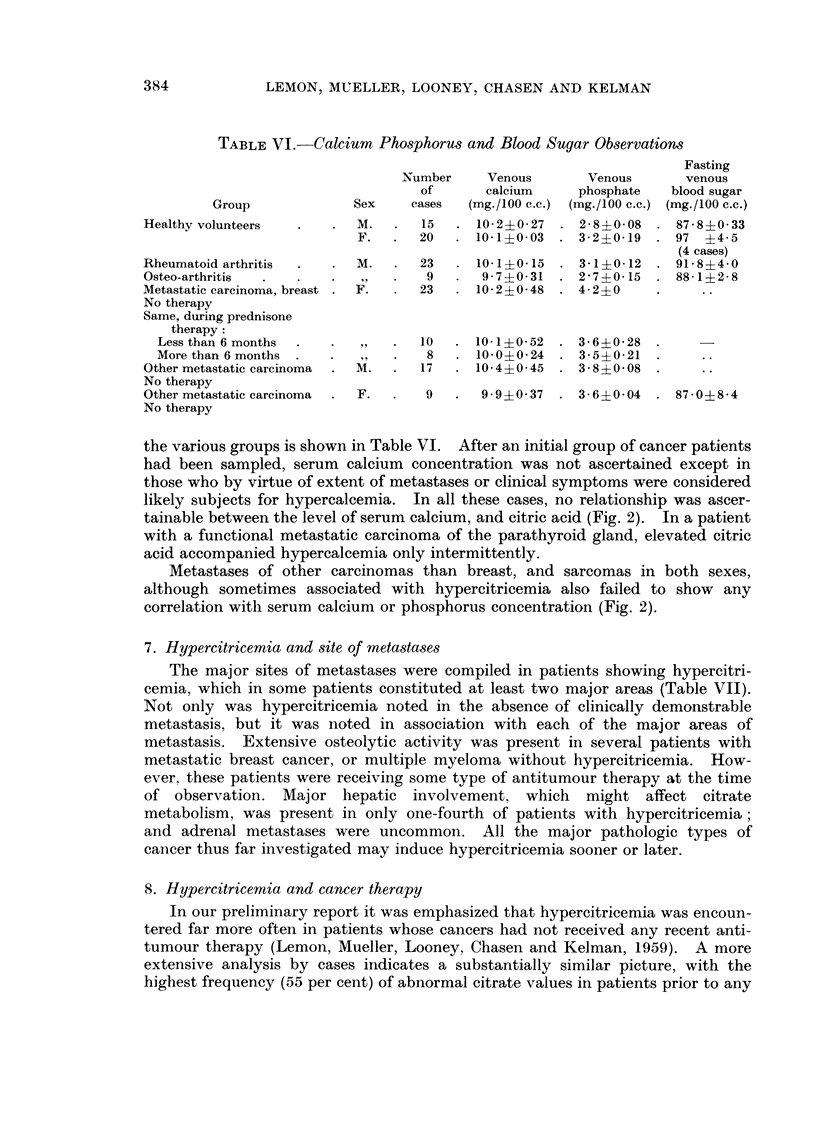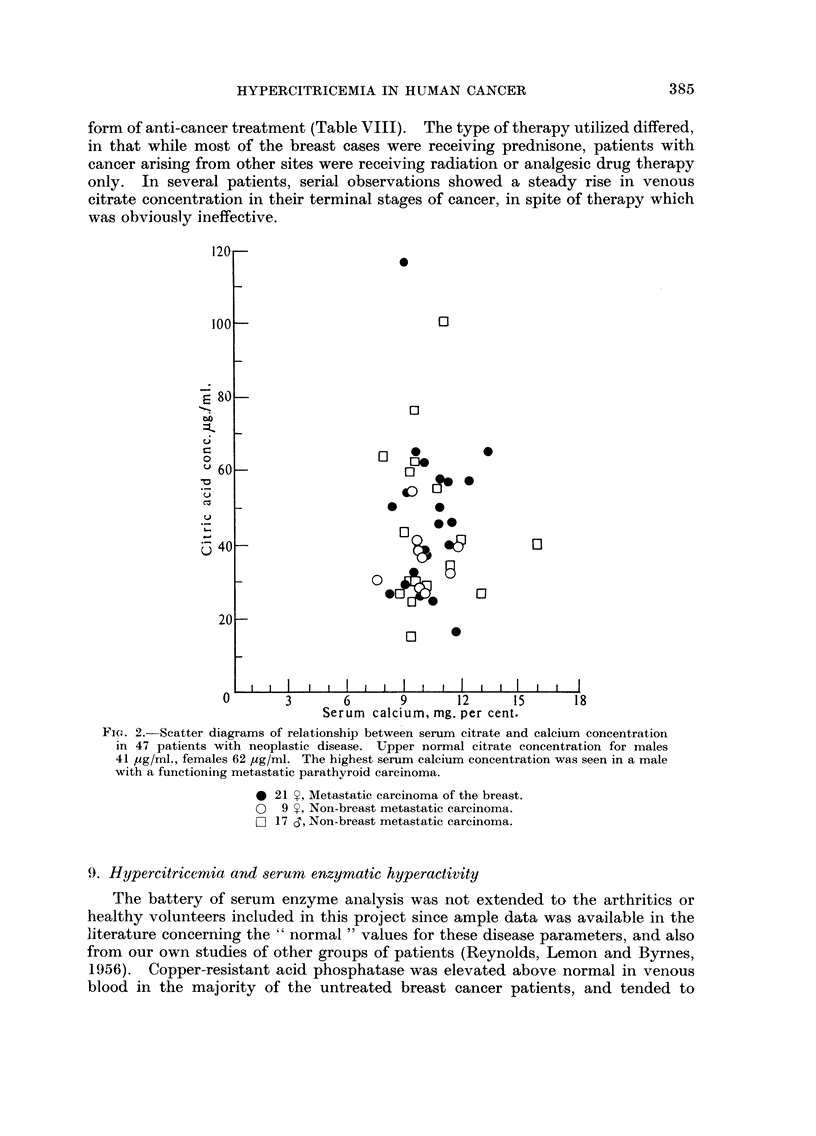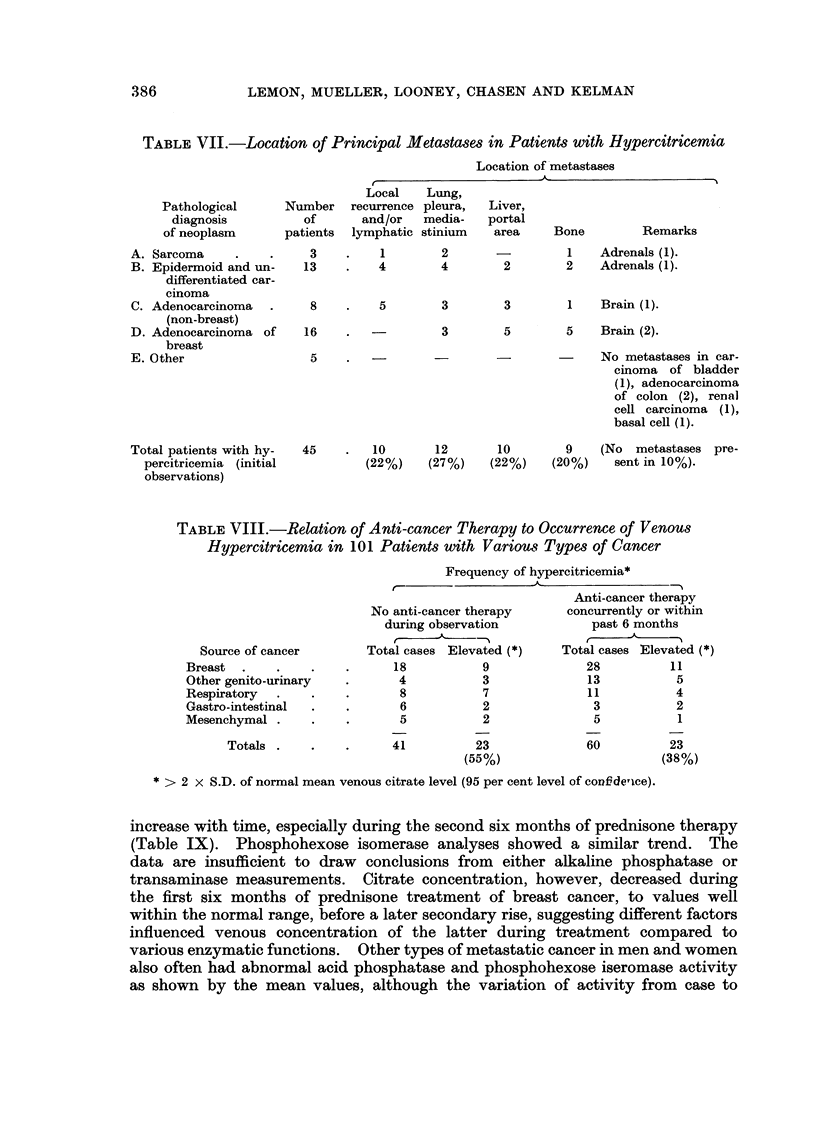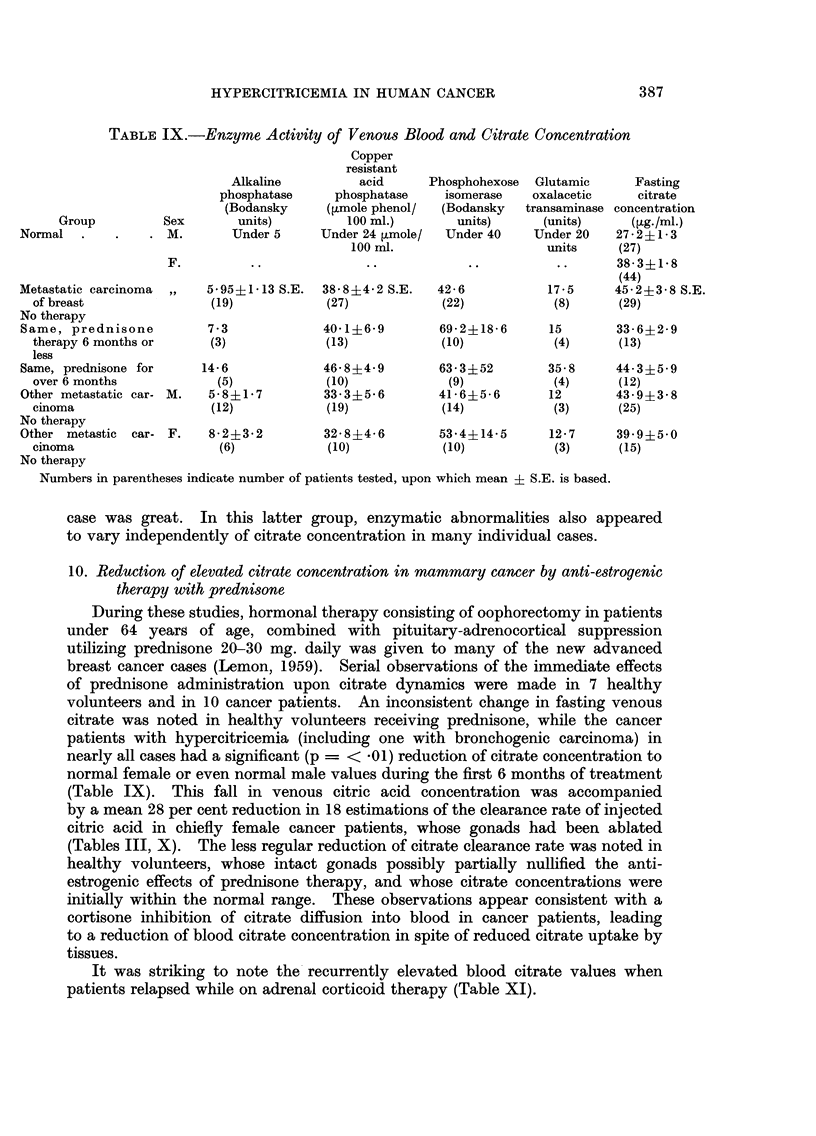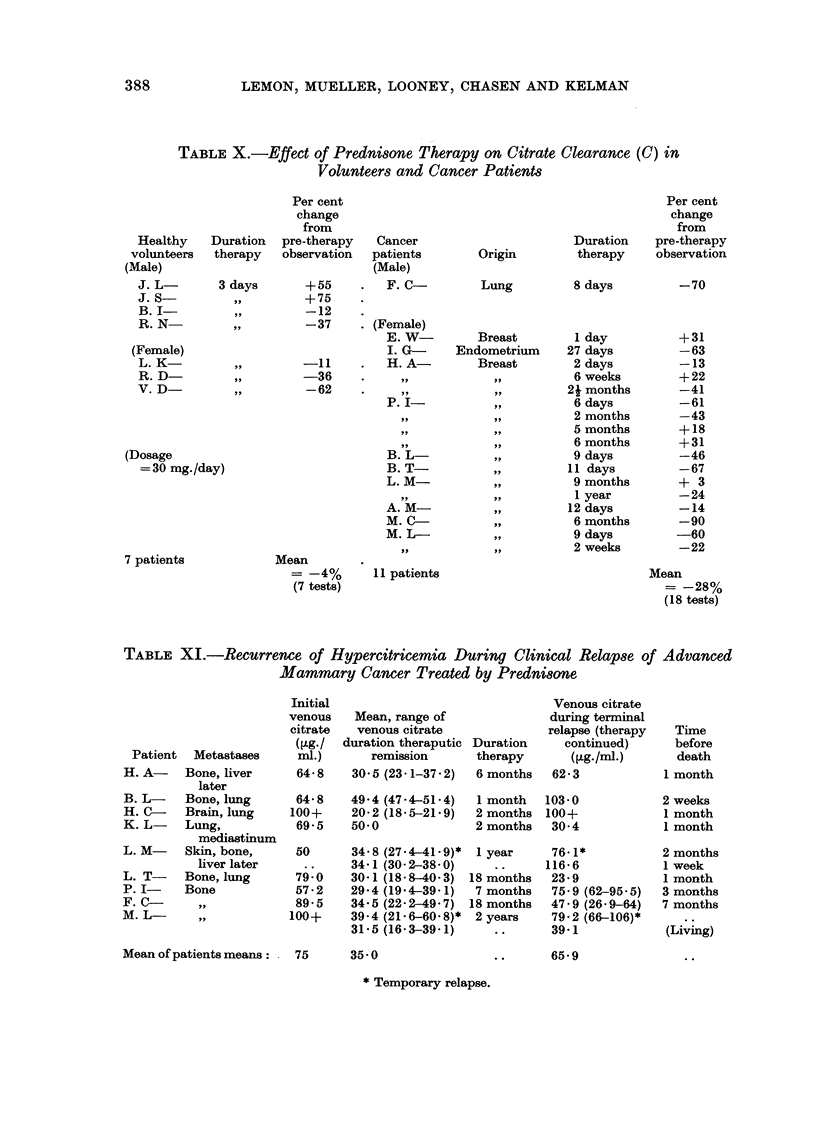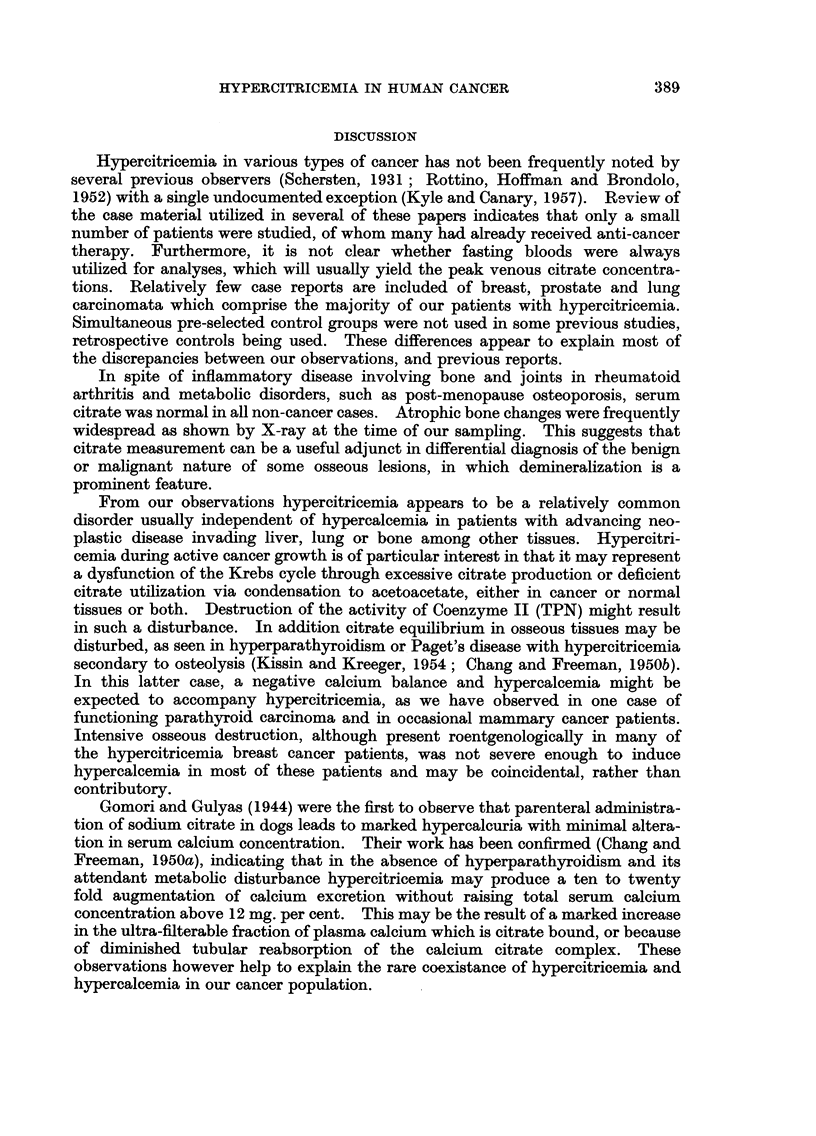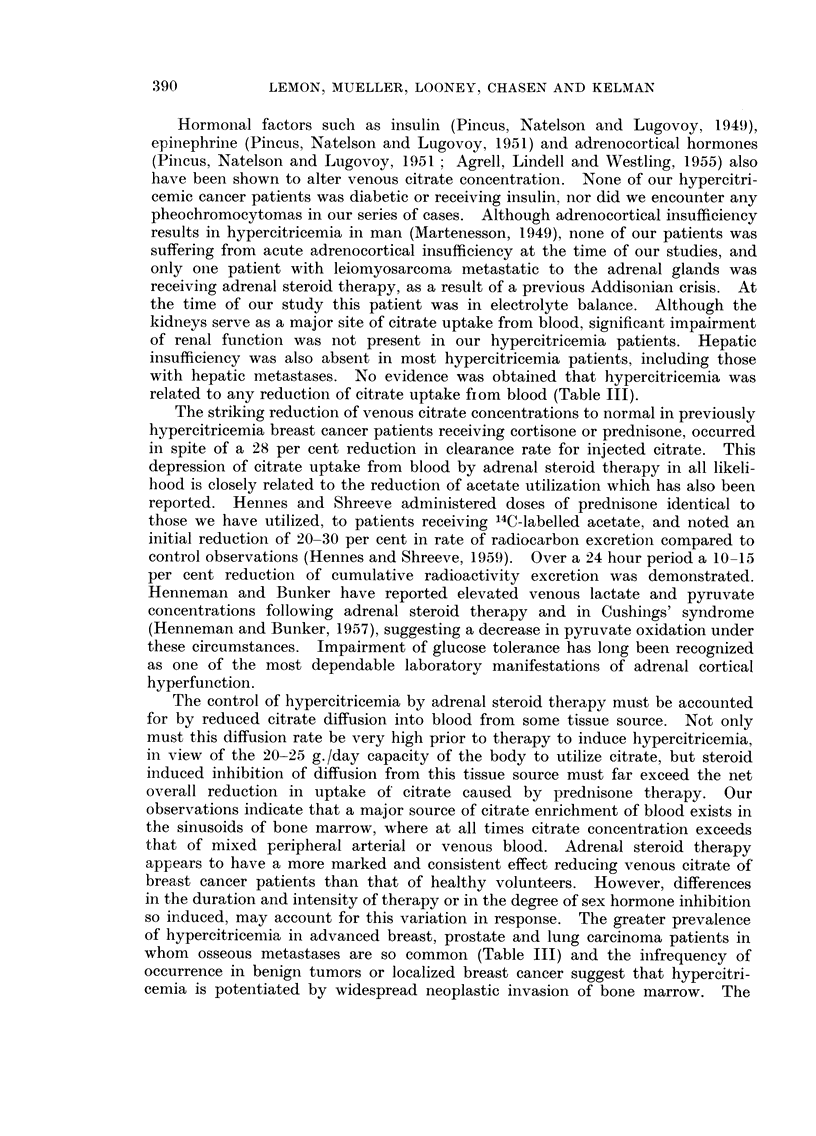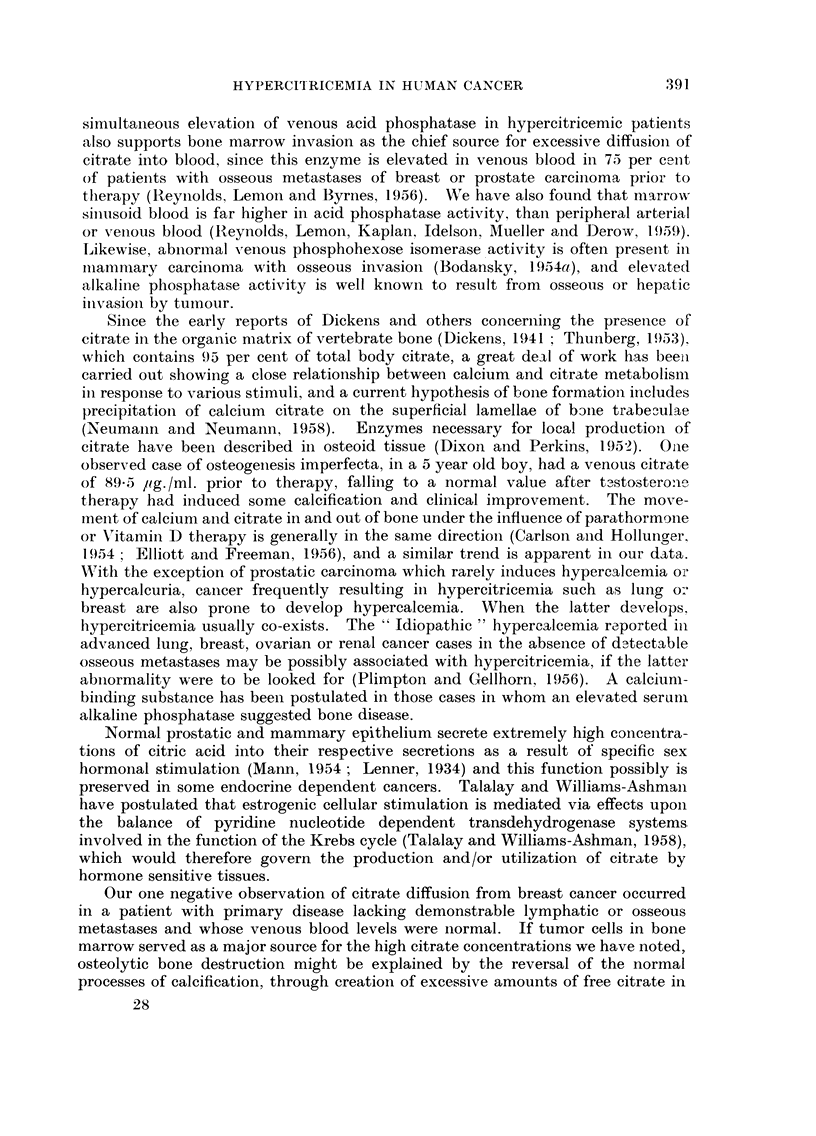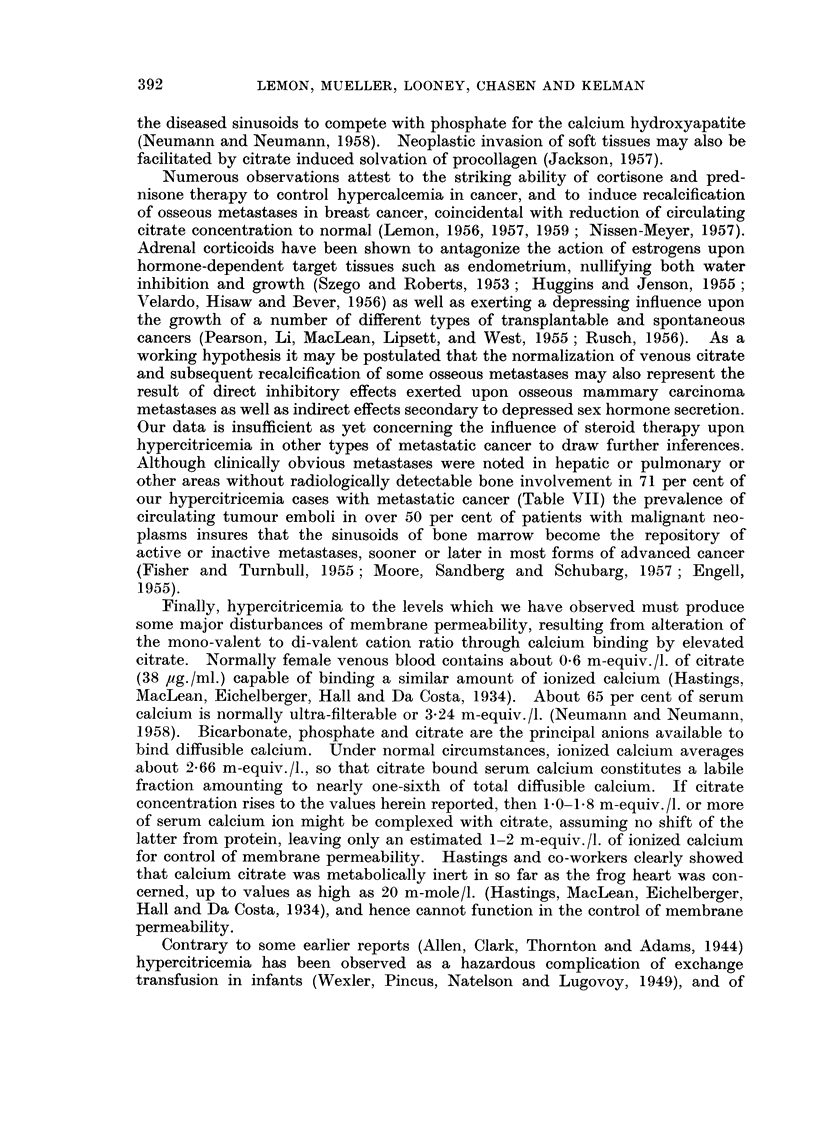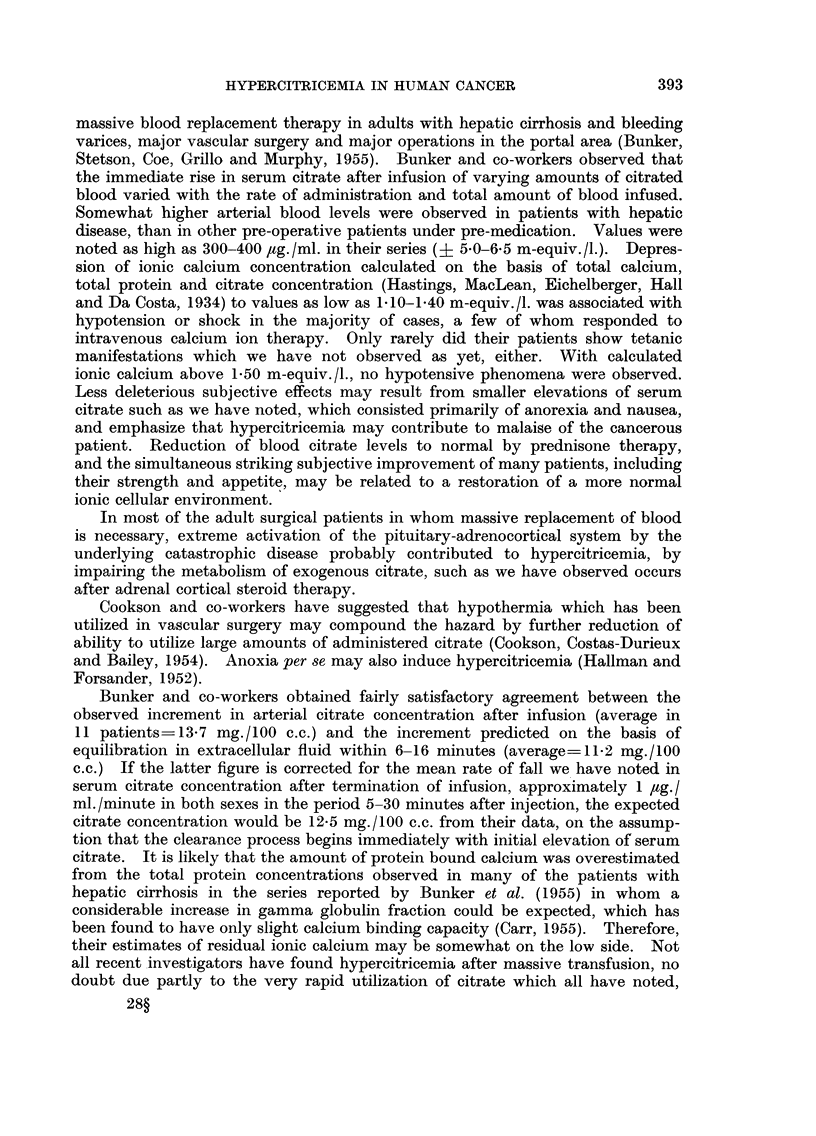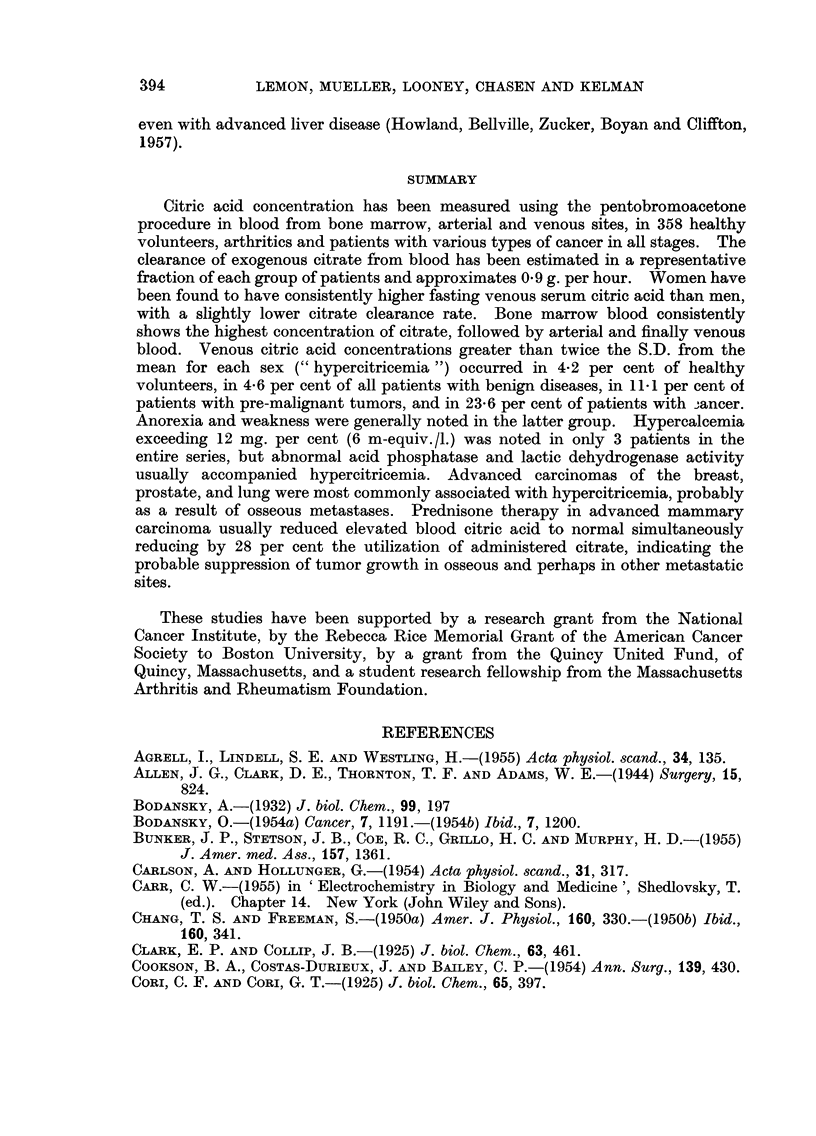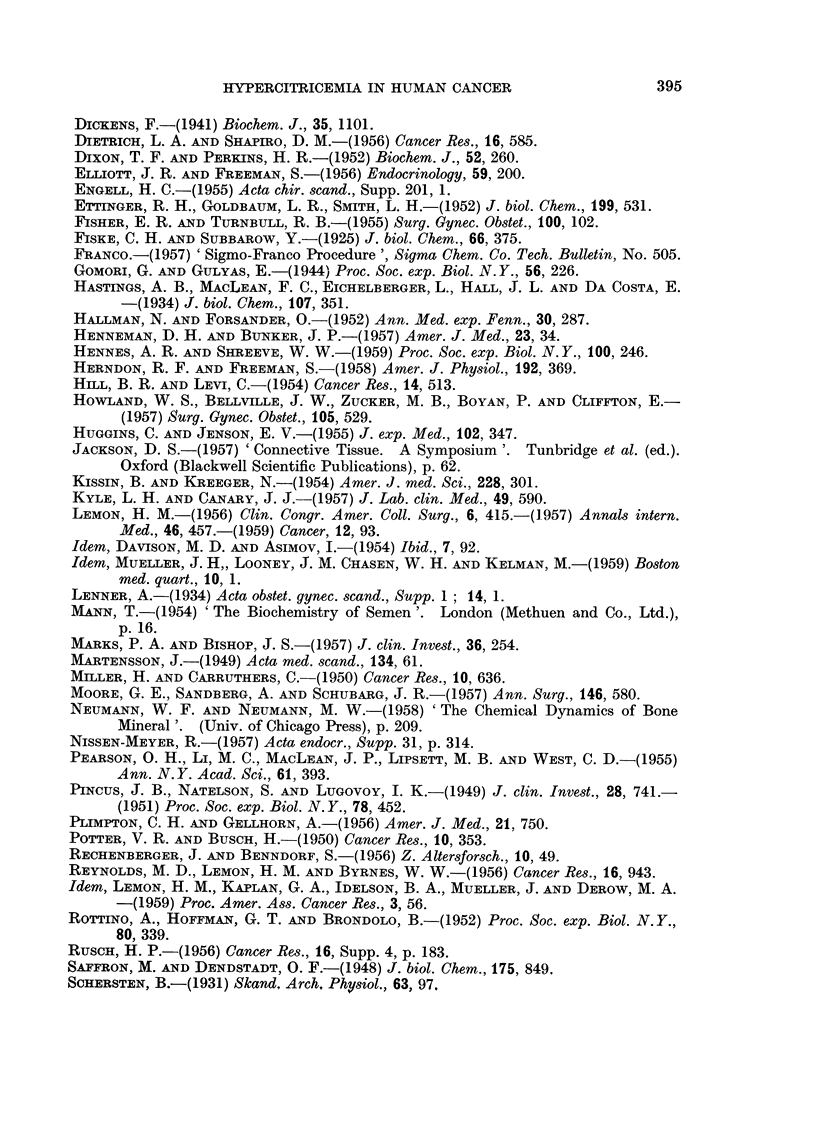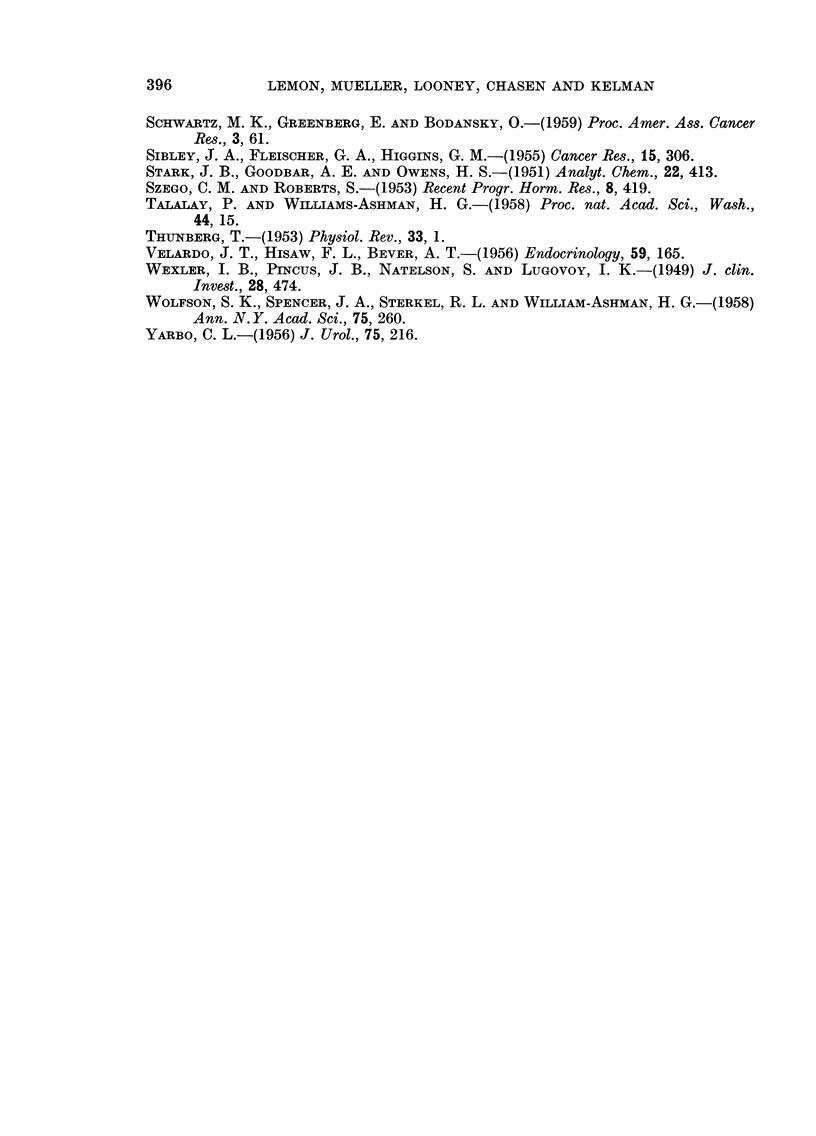# Hypercitricemia in Human Cancer. Factors concerned in Pathogenesis and Treatment

**DOI:** 10.1038/bjc.1960.44

**Published:** 1960-06

**Authors:** H. M. Lemon, J. H. Mueller, J. M. Looney, W. H. Chasen, Marcia Kelman


### HYPERCITRICEMIA IN HUMAN CANCER FACTORS CONCERNED IN PATHOGENESIS AND TREATMENT

H. M. LEMON,* J. H. MUELLER,† J. M. LOONEY, W. H. CHASEN AND MARCIA KELMAN

*From the Division of Neoplastic Disease, Department of Medicine, the Department of Biochemistry, Boston University School of Medicine and the Outpatient Clinic, Boston Veterans Administration, Massachusetts, U.S.A.*

Received for publication January 12, 1960

PATIENTS with leukemia, metastatic carcinoma and sarcoma possess altered carbohydrate metabolism as shown by reduced glucose tolerance (Marks and Bishop, 1957), and elevated resting venous lactic acid (Cori and Cori, 1925). Enzymes concerned in tissue glycolysis are often increased in activity in serum during neoplastic disease progression, including phosphohexose isomerase (Bodansky, 1954*b*), aldolase (Sibley Fleischer and Higgins, 1955), and lactic dehydrogenase (Hill and Levi, 1954). Acid phosphomonoesterases hydrolyzing three-carbon substrates produced during glycolysis are also increased in activity in venous blood from patients with breast and prostate cancer (Reynolds, Lemon and Byrnes, 1956). Observations indicating possible abnormalities of the Krebs tricarboxylic acid cycle in human cancer patients however are limited to increased DPN-dependent dehydrogenase activity in sera of patients with hepatic metastases (Wolfson, Spencer, Sterkel, and Williams-Ashman, 1958; Schwartz, Greenberg, and Bodansky, 1959), and a single report of abnormal venous citric acid (Kyle and Canary, 1957). In this communication we are reporting hypercitricemia as a frequent abnormality of untreated advanced cancer. A preliminary account of these observations has been published elsewhere (Lemon, Mueller, Looney, Chasen and Kelman, 1959).

### METHODS

Citrate concentration in blood obtained from various sites has been determined utilizing the Ettinger modification of the highly specific pentobromoacetone method (Ettinger, Goldbaum and Smith, 1952). Blood samples were obtained from volunteers and patients following a 12-hour fast and analyses performed in duplicate in most instances. Chloral hydrate which is the only known drug possibly reacting to this proceedure has not been administered within 12 hours to most of the patients or any of the volunteers studied. As a further check on the methods used, citric and other organic acids have been separated and concentrated from sera by a method developed by Dr. H. H. Wotiz, using 4N KOH to precipitate serum proteins followed by neutralization to pH 6·0 with concentrated HCl. The precipitate was centrifuged and washed once with distilled $H_2O$. The washings combined with the supernate were made slightly alkaline

* Present address: Massachusetts Memorial Hospitals, 750 Harrison Avenue, Boston 18, Massachusetts.

† Present address: National Naval Medical Center, Bethesda, Maryland.

with 2·5 N NaOH, and reacidified by bubbling $CO_2$ through the solution. These extracts were evaporated to dryness, resuspended in 1·0 c.c. ethanol and then chromatographed, along with reference citrate standards (Stark, Goodbar, and Owens, 1951). The citrate zone was then eluted, and the Ettinger procedure applied to the extract. A close agreement with the original analysis of unextracted serum was usually obtained in sera from cancerous and non-cancerous patients, indicating that citrate rather than some other organic acid was actually being measured.*

Blood was obtained in some volunteers and patients by simultaneous posterior iliac crest aspiration, arterial puncture and venipuncture. Following the initial sample 0·5 g. of sodium citrate as a 4 per cent solution was injected intravenously in a two minute period in many patients, with additional venous samples obtained at 5, 10, 20 and 30 minutes following commencement of injection. Since it was soon found that citrate concentration decreased rapidly with time between 5–30 minutes after injection, comparable to a first order reaction (Lemon, Mueller, Looney, Chasen and Kelman, 1959), only 5 and 30 minute samples were routinely collected for measurement of clearance rates. Analysis of serum calcium (Clark and Collip, 1925), phosphorus (Fiske and Subbarow, 1925) and glucose before and 30 minutes following injection was performed on a sample of 40 patients. Periodic serum calcium, phosphorus and alkaline phosphatase (Bodansky, 1932) analyses were performed in addition on most of the cancer patients. Simultaneous observations of copper-resistant serum acid phosphatase, (Reynolds, Lemon and Byrnes, 1956) and glutamic oxalacetic transaminase, (Franco, 1957) were made on the same serum sample, in a representative series comprising many of the cancer patients.

Patients were classified according to the nature of their principal disease. Biopsy proof of cancer was obtained in all the cancer patients investigated, and the extent and location of their metastases was assessed from clinical and radiologic diagnostic procedures. Table I summarizes the diagnostic information on the patients studied.

In the statistical evaluation of data, only the initial serum-observation was utilized for the comparison of means between different groups of patients with various diseases. This tends to underestimate the frequency of elevated blood citric acid, which is more frequent in the more advanced stages of cancer, but is necessary for a true comparison between cancer and other diseases, in which only a single observation was available for each case. Hypercitricemia has been defined as serum citrate value exceeding twice the standard deviation of the mean of healthy volunteers of the same sex, a value which is at the 95 per cent level of confidence for abnormality.

### RESULTS

### 1. *Citrate dynamics*

The increase of serum citrate from 0–5 minutes ($\triangle$) was regarded as the result of rapid dilution of the 0·5 g. dose into plasma and extracellular fluid, an

* The analytic procedure has repeatedly yielded 95–98 per cent recovery of citrate added to whole blood or serum. Excellent checks within 2–3 per cent accuracy have been obtained when duplicate analyses for citrate were carried out using the method reported by Saffron and Dendstadt (1948). All sera were immediatley frozen upon separation from clot and analyses should be carried out within a few days of collection. Frozen sera may show 5–10 per cent changes in citrate concentration with prolonged storage. Simultaneous citrate standards were routinely run with each group of analyses.

TABLE I.—*Classification of Clinical Material*

| Diagnosis | Source of material | Number of patients | Nutrition | Stage of Disease |
|---|---|---|---|---|
| Healthy volunteers . | Medical students. Hospital personnel | 71 | Excellent | — |
| Rheumatoid and degenerative arthritis | Ambulatory out-patients, with 15-year documented history | 32 | Good | Stage II. III. American Rheumatism Association. |
| Non-cancer disorders . | Chiefly hospital patients, some bed-fast | 33 | Good to fair | — |
| Pre-malignant lesions and benign tumours | Ambulatory out-patients and hospital in-patients | 28 | Excellent to good | — |
| Carcinoma and sarcoma | Ambulatory and bed-fast hospital patients | 195 | Good to fair | All stages, from early localized surgically cured to distant metastases. |
| Total . . | — | 358 | — | — |

assumption which appears valid (Bunker, Stetson, Coe, Grillo, and Murphy, 1955). This distribution space S was determined by dividing the total in $\mu$g. ($0{\cdot}5 \times 10^6$) by $\triangle$ given in $\mu$g. per ml. In healthy volunteers and cancer patients the mean for this space was 21·6 liters for males and 14·0 liters for females (Table II).

Following the peak concentration of citrate 5 minutes after start of injection serum citrate concentration rapidly declined in a semilogarithmic manner compatible with a first order reaction, the initial baseline concentration very nearly being attained in normal, arthritic and cancer patients at 30 minutes time (Lemon, Mueller, Looney, Chasen and Kelman, 1959). The rate of decrease of serum citrate concentration approximated 1 $\mu$g./ml./min., which when multiplied by $S \times 60$ in each case provided an estimate of the hourly rate of citrate clearance from plasma and extracellular fluid, by diffusion, metabolism, and renal excretion. In normal males, plasma clearance approximated 900 mg. per hour and in females, 800 mg. per hour. Urinary citrate excretion was not measured, since preliminary studies showed poor correlation between serum and urine citrate concentration. Citrate excretion in the urine appears to fluctuate quite independently of serum values owing to high renal uptake and metabolism (Herndon and Freeman, 1958). Only a small fraction of plasma citrate passing through the kidney is excreted, the amount being affected by acid-base balance and vitamin D content of the diet (Yarbo, 1956).

2. *Fasting venous citrate concentrations and disease*

In conformity with observations published by Rechenberger and Benndorf while this study was underway (Rechenberger and Benndorf, 1956), the mean venous citrate concentration of healthy females was found to exceed that of males of comparable age groups (Fig. 1 ; Tables II, III). The mean fasting venous concentration of male and female patients with rheumatoid and osteoarthritis was almost identical to that of their sex-matched volunteer controls.

Patients with benign tumours such as benign prostatic hyperplasia and mammary dysplasia had citrate concentrations also within the normal range for their

TABLE II.—*Observations of Citrate Dynamics in Healthy Volunteers and Patients with Benign Diseases*

| Group | Sex | Number of cases | Mean fasting venous citrate concen-tration | Range | Frequency of observation above 2×S.D. | Distribution space (liters ±S.E.) | Clearance rate (mg./hr. ±S.E.) |
|---|---|---|---|---|---|---|---|
| *Normal* | | | | | | | |
| Volunteers | M. | 27 | 27·2±1·3 ($p$<0·001)* | 16·4–45·7 | 1 | 21·6±2·4 ($p$=0·012)* | 896±58 |
| | F. | 44 | 38·3±1·8 | .. | 2 | 14·9±1·3 | 799±38 |
| | | 71 | | | 3=4·2% | | |
| *Non-cancer* | | | | | | | |
| Hepatic cirrhosis | M. | 3 | 33·9 | 18–61·1 | 1 | .. | .. |
| | F. | 4 | 46·5 | 34·8–51·0 | 0 | .. | .. |
| Osteo-porosis | ,, | 2 | .. | 20·2–38·4 | 0 | .. | .. |
| Pregnancy | ,, | 3 | 25·0 | 14·4–31·0 | 0 | .. | .. |
| Other disease | M. | 10 | 34·5±5·7 | 14·8–84·9 | 1 | .. | .. |
| | F. | 10 | 39·1±4·8 | 20·4–64·4 | 1 | .. | .. |
| Rheumatoid arthritis | M. | 23 | 27·9±1·2 | 20–39 | 0 | 31·4±4·8 | 1027±33 |
| Osteo- and degenerative arthritis | ,, | 9 | 26·5±0·4 | 23·5–30·5 | 0 | 26·5±2·1 | 955±71 |
| | | 65 | | | 3=4·6% | | |
| *Pre-malignant* | | | | | | | |
| Mammary dysplasia | F. | 13 | 30·4±2·7 | 16·1–49·9 | 0 | .. | .. |
| Gynecomastia | M. | 2 | — | 24·0–58·5 | 1 | .. | .. |
| Benign prostatic hyperplasia | ,, | 12 | 29·0±2·6 | 15·7–44·8 | 2 | .. | .. |
| | | 27 | | | 3=11·1% | | |
| Grand total | .. | 163 | .. | .. | 9=5·5% | .. | .. |

* Significance between upper and lower figures.

sex. However, a few patients with carcinomas amenable to surgical excision and with clinically detectable metastases showed abnormal elevation of fasting venous citrate concentrations (Table III).

Women of all age groups with untreated metastatic carcinoma of the breast, had fasting venous citrate concentrations significantly (p = < ·01) in excess of volunteers, arthritic females or women with mammary dysplasia (Tables II, IV). Concentrations of venous citrate often exceeded more than 57 $\mu$g./ml. in untreated patients with advanced disease or patients in terminal relapse following a hormone-induced disease remission. Equally high citrate concentrations were noted in male patients with a variety of other types of metastatic carcinoma, including bronchogenic and pulmonary carcinoma, gastrointestinal carcinoma, fibrosarcoma, and lymphoma (Table III). Hypercitricemia of this degree has been noted only rarely in non-cancer patients, including one obese female with mild uncontrolled diabetes mellitus, an anxious male with questionable peptic ulcer symptoms and negative X-ray findings, and one case of osteogenesis imperfecta.

FIG. 1.—Mean fasting serum citric acid concentration in healthy volunteer controls and untreated metastatic breast cancer in females, grouped by age decade. Small numbers at each point indicate number of patients observed. Horizontal dashed line indicates mean, with range of standard error indicated by upright bracket at left, for the entire series of patients in each of the three classifications.

●———● Untreated metastatic breast cancer (24).

*Controls*

○———○ Female (55).

□———□ Male (37).

The discrepancy between the total cases and the sums at each point in this chart represents those rare cases for whom age was unknown from available records and solitary cases of other age decade categories which were not suitable for plotting as means.

3. *Citrate clearance rate and disease*

No significant sex difference was noted in mean citrate clearances (Table II). Male patients with extensive rheumatoid arthritis cleared injected citrate from their blood stream at rates which did not differ significantly from male or female normal volunteers. There was no significant deviation from volunteers in the average rate of citrate clearance by patients with various cancers other than breast, except for males with inactive post-therapy non-breast cancer, who showed a subnormal mean clearance rate in the small group tested (Table III). However, untreated patients with disseminated breast cancer tended to have the highest mean rate of citrate clearance, of any of the groups studied, as well as the highest

TABLE III.—*Observations of Citrate Dynamics in Patients with Cancer*

| Group | Sex | Number of patients | Mean fasting venous citrate (μg/ml. + S.E. initial observation) | Range | Frequency of observations exceeding 2 × S.D. | Number of patients | Distribution space (liters ± S.E.) | Clearance rate (mg./hr. + S.E.) |
|---|---|---|---|---|---|---|---|---|
| *Non-breast Cancer* | | | | | | | | |
| Local, pre-operative . | M. | 5 | 36·3±5·6 | 15·7–50 | 3 | .. | .. | .. |
| | F. | 1 | .. | 72·5 | 1 | .. | .. | .. |
| Inactive post-therapy | M. | 7 | 36·0±4·3 | 16·9–52·9 | 2 | 8 | 18·7±3·6 | 490±107 |
| | F. | 9 | 38·8±7·2 | 16·9–76·1 | 3 | 6 | 19·4±4·1 | 1013± 90 |
| Active distant metastases | M. | 42 | 42·0±3·2 | 15·5–100 | 14 | 12 | 15·8±2·1 | 828±127 |
| | F. | 35 | 35·7±2·2 | 12·1–69·9 | 3 | 8 | 18·2±2·6 | 715±130 |
| Carcinoma of prostate | M. | 8 | 37·6±5·9 | 14·9–66·5 | 3 | .. | .. | .. |
| | | 107 | | | 29=27% | | | |
| *Carcinoma of Breast* | | | | | | | | |
| Local, pre-operative . | F. | 6 | 27·8±2·7 | 20·7–39·5 | 0 | .. | .. | .. |
| Inactive, post-therapy | ,, | 20 | 36·5±1·7 | 22·3–51·4 | 0 | .. | .. | .. |
| Active distant metastases no therapy | ,, | 41 | 52·1±3·5 | 19·4–116·6 | 11 | 18 | 18·2±2·7 | 992 ±72 |
| Active metastases treated by sex hormones | ,, | 3 | .. | 22·9–72·6 | 1 | .. | .. | ($p$<0·01)* |
| Active metastases treated by cortisone or prednisone | ,, | 18 | 46·2±5·6 | 19·4–93·0 | 4 | 26 | 15·5±1·5 | 720± 59 |
| | | 88 | | | 16=18·4% | | | |
| Grand total . | | 195 | | | 45=23% | 78 | | |

* Significance of difference between upper and lower figures.

mean fasting venous citrate. Following therapy with adrenal corticoid hormones, breast cancer patients showed a reduction of citrate clearance rates, as well as a reduction in fasting citrate level.

In one patient with breast cancer, citrate clearance was simultaneously measured in mixed venous blood, and in venous blood passing through the primary tumour. The citrate clearance rate was identical in both areas, according to our method of calculation (Table IV). This suggests that the difference in blood citrate concentration in the two sites was not due to uptake of citrate by the neoplasm.

These results would suggest increased citrate diffusion from some body tissue accounting for the increased blood level, rather than reduced tissue utilization in carcinomatosis. Bone which contains over 1 per cent citrate (dry weight), in its organic matrix (Dickens, 1941 ; Thunberg, 1953) is one of the more obvious sources for citrate release, but metastatic cancer and malignant tumour cells themselves contain considerable amounts of citrate (Potter and Busch, 1950 ; Dietrich and Shapiro, 1956 ; Miller and Carruthers, 1950).

4. *Citrate distribution space and disease*

A sex difference was noted in the mean distribution space of healthy volunteers, which was statistically significant at a level of confidence (p $=\pm$ ·012 ; Table II).

TABLE IV.—*Acid Phosphatase Activity and Citrate Concentration in Venous Blood Draining Breast Cancer Primary Site, Compared to Mixed Venous Blood*

| | | Venous blood from left breast primary tumor | | Mixed venous blood from right antecubital vein | |
|---|---|---|---|---|---|
| Date | Time | Citrate (µg./ml.) | Acid phosphatase (µmole/100 ml.) | Citrate (µg./ml.) | Acid phosphatase (µmole/100 ml.) |
| 1/13/58 | Fasting | 15 | 27·6 | 26·9 | 13·6 |
| | 5 minutes after citrate injection | 23·5 | 39·1 | 70·0 | 30·2 |
| | 30 minutes after citrate injection | — | 25·8 | 34·5 | 16·4 |
| 1/16/59 | Fasting | 16·5 | 29·4 | 33·0 | 25·6 |
| | 7 minutes after injection | — | — | 65·5 | 18·8 |
| | 10 minutes after injection | 33·5 | 27·2 | — | — |
| | 26 minutes after injection | — | — | 35·5 | 24·5 |
| | 29 minutes after injection | 19·5 | 28·3 | — | — |
| Mean values | | 21·6 | 29·6 | 44·2 | 21·5 |
| Citrate dynamics 1/13/58 | Distribution space | — | | 11·6 liters | |
| | Clearance rate | — | | 990 mg./hr. | |
| 1/16/58 | Distribution space | 29·4 liters | | 13·7 liters | |
| | Clearance rate | 1300 mg./hr. | | 1300 mg./hr. | |

A sex difference was not seen however in either of two groups of non-breast cancer patients, in whom the average male distribution space approached the female value. Male rheumatoid arthritic patients, on the other hand possessed a higher mean distribution space for citrate than healthy volunteers, and were significantly different in this respect from all groups of male or female patients with active metastatic cancer of breast as well as other types of cancer ($p = < \cdot 012$). These results suggest that the tissues of patients with rheumatoid arthritis are more widely and rapidly permeable to injected citrate resulting in a 5 minute post-injection peak value which is considerably smaller than observed in patients with cancer, or healthy volunteers, as a result of dilution. This observation may be related to the unusual property of citric acid in solubilizing pro-collagen (Jackson, 1957).

5. *Variations in citrate concentration in blood obtained from various sites*

Citric acid obviously is in a state of rapid flux in the blood, with removal rates in the vicinity of 0·8–0·9 g./hr. which must be matched by release of citrate into blood at an equivalent rate. In an effort to learn more about this phase of citrate metabolism, venous samples were obtained from blood leaving a large mucoid adenocarcinoma of the breast, and compared to mixed venous blood from an antecubital vein :

*ABSTRACT*

Case No. 1. This Patent, F. W., aged 65, Boston City Hospital No. 1630205, had a 11 × 15 × 15 cm. untreated neoplasm of the left breast reported as mucin secreting adenocarcinoma by biopsy. Two large superficial veins were noted draining the massive primary lesion, passing from the mid left thorax upward to anastomose with communicating branches to the left internal mammary vein. Two citrate tolerance tests were carried out, 1/13/58 and 1/16/58, before and after 30 mg. daily of prednisone therapy, starting 1/14/58 (Table IV). A radical mastectomy on the left was performed on 1/20/58, with 8 negative lymph nodes found. A right radical mastectomy was performed on 1/31/58 for a simultaneous primary on the other side, measuring 1·0 × 1·4 × 1·0 cm., reported as medullary and scirrhus carcinoma, also with negative lymph nodes.

The results demonstrated clearly a decreased citrate concentration in blood leaving this particular cancer, although acid phosphatase activity, which is believed to diffuse from malignant tumours, (Lemon, Davison, and Asimov, 1954), was definitely increased. The latter observation confirms that we were examining a blood sample in the tumour venous bed which differed significantly in its properties from mixed antecubital venous blood, in all six samples. As a result of this observation, some other major source than tumour tissue for citrate diffusion into blood had to be postulated to explain the elevated serum citrate noted in breast and other cancer patients. Simultaneous blood samples from iliac bone marrow, brachial or radial artery, and antecubital vein were then obtained, in a series of healthy volunteers, and cancer patients. A uniform decreasing gradient of citrate concentration from marrow to artery to vein was observed both in representative normal individuals and in cancer patients (Table V).

TABLE V.—*Relative Concentration of Citric Acid in Blood from Various Sites*

| | | | Mean citrate concentration | | |
|---|---|---|---|---|---|
| Group | No. | Sex | Marrow blood ($\mu$g./ml.) | Arterial blood (as % of marrow concentration) | Venous blood (as % of marrow concentration) |
| Healthy volunteers | 11 | M. | 34·1 | 89 (4 cases) (range 81–94) | 73 (range 51–92) |
| Metastatic cancer patients (6 under treatment) | 8 | F. | 45·0 | 93 (range 87–98) | 86 (range 67–95) |

6. *Serum calcium and phosphorus concentration*

The injection of 0·5 g. of sodium citrate did not significantly change serum calcium, phosphorus or glucose concentration in a sample of 40 patients within the 30 minute period of observation. Hypercalcemia above 12·0 mg. per cent was noted in only one out of 45 patients with active metastatic breast cancer in this study, and in only two out of 85 patients with other types of metastatic cancer. One of the latter cases was a functioning parathyroid carcinoma. The mean serum calcium and phosphorus concentrations for patients sampled within

TABLE VI.—*Calcium Phosphorus and Blood Sugar Observations*

| Group | Sex | Number of cases | Venous calcium (mg./100 c.c.) | Venous phosphate (mg./100 c.c.) | Fasting venous blood sugar (mg./100 c.c.) |
|---|---|---|---|---|---|
| Healthy volunteers | M. | 15 | 10·2±0·27 | 2·8±0·08 | 87·8±0·33 |
| | F. | 20 | 10·1±0·03 | 3·2±0·19 | 97 ±4·5 (4 cases) |
| Rheumatoid arthritis | M. | 23 | 10·1±0·15 | 3·1±0·12 | 91·8±4·0 |
| Osteo-arthritis | ,, | 9 | 9·7±0·31 | 2·7±0·15 | 88·1±2·8 |
| Metastatic carcinoma, breast No therapy | F. | 23 | 10·2±0·48 | 4·2±0 | .. |
| Same, during prednisone therapy : | | | | | |
| Less than 6 months | ,, | 10 | 10·1±0·52 | 3·6±0·28 | — |
| More than 6 months | ,, | 8 | 10·0±0·24 | 3·5±0·21 | .. |
| Other metastatic carcinoma No therapy | M. | 17 | 10·4±0·45 | 3·8±0·08 | .. |
| Other metastatic carcinoma No therapy | F. | 9 | 9·9±0·37 | 3·6±0·04 | 87·0±8·4 |

the various groups is shown in Table VI. After an initial group of cancer patients had been sampled, serum calcium concentration was not ascertained except in those who by virtue of extent of metastases or clinical symptoms were considered likely subjects for hypercalcemia. In all these cases, no relationship was ascertainable between the level of serum calcium, and citric acid (Fig. 2). In a patient with a functional metastatic carcinoma of the parathyroid gland, elevated citric acid accompanied hypercalcemia only intermittently.

Metastases of other carcinomas than breast, and sarcomas in both sexes, although sometimes associated with hypercitricemia also failed to show any correlation with serum calcium or phosphorus concentration (Fig. 2).

7. *Hypercitricemia and site of metastases*

The major sites of metastases were compiled in patients showing hypercitricemia, which in some patients constituted at least two major areas (Table VII). Not only was hypercitricemia noted in the absence of clinically demonstrable metastasis, but it was noted in association with each of the major areas of metastasis. Extensive osteolytic activity was present in several patients with metastatic breast cancer, or multiple myeloma without hypercitricemia. However, these patients were receiving some type of antitumour therapy at the time of observation. Major hepatic involvement, which might affect citrate metabolism, was present in only one-fourth of patients with hypercitricemia ; and adrenal metastases were uncommon. All the major pathologic types of cancer thus far investigated may induce hypercitricemia sooner or later.

8. *Hypercitricemia and cancer therapy*

In our preliminary report it was emphasized that hypercitricemia was encountered far more often in patients whose cancers had not received any recent antitumour therapy (Lemon, Mueller, Looney, Chasen and Kelman, 1959). A more extensive analysis by cases indicates a substantially similar picture, with the highest frequency (55 per cent) of abnormal citrate values in patients prior to any form of anti-cancer treatment (Table VIII). The type of therapy utilized differed, in that while most of the breast cases were receiving prednisone, patients with cancer arising from other sites were receiving radiation or analgesic drug therapy only. In several patients, serial observations showed a steady rise in venous citrate concentration in their terminal stages of cancer, in spite of therapy which was obviously ineffective.

FIG. 2.—Scatter diagrams of relationship between serum citrate and calcium concentration in 47 patients with neoplastic disease. Upper normal citrate concentration for males 41 μg/ml., females 62 μg/ml. The highest serum calcium concentration was seen in a male with a functioning metastatic parathyroid carcinoma.

● 21 ♀, Metastatic carcinoma of the breast.

○ 9 ♀, Non-breast metastatic carcinoma.

□ 17 ♂, Non-breast metastatic carcinoma.

9. *Hypercitricemia and serum enzymatic hyperactivity*

The battery of serum enzyme analysis was not extended to the arthritics or healthy volunteers included in this project since ample data was available in the literature concerning the " normal " values for these disease parameters, and also from our own studies of other groups of patients (Reynolds, Lemon and Byrnes, 1956). Copper-resistant acid phosphatase was elevated above normal in venous blood in the majority of the untreated breast cancer patients, and tended to

TABLE VII.—*Location of Principal Metastases in Patients with Hypercitricemia*

| Pathological diagnosis of neoplasm | Number of patients | Location of metastases: Local recurrence and/or lymphatic | Lung, pleura, mediastinium | Liver, portal area | Bone | Remarks |
|---|---|---|---|---|---|---|
| A. Sarcoma | 3 | 1 | 2 | — | 1 | Adrenals (1). |
| B. Epidermoid and undifferentiated carcinoma | 13 | 4 | 4 | 2 | 2 | Adrenals (1). |
| C. Adenocarcinoma (non-breast) | 8 | 5 | 3 | 3 | 1 | Brain (1). |
| D. Adenocarcinoma of breast | 16 | — | 3 | 5 | 5 | Brain (2). |
| E. Other | 5 | — | — | — | — | No metastases in carcinoma of bladder (1), adenocarcinoma of colon (2), renal cell carcinoma (1), basal cell (1). |
| Total patients with hypercitricemia (initial observations) | 45 | 10 (22%) | 12 (27%) | 10 (22%) | 9 (20%) | (No metastases present in 10%). |

TABLE VIII.—*Relation of Anti-cancer Therapy to Occurrence of Venous Hypercitricemia in* 101 *Patients with Various Types of Cancer*

| | Frequency of hypercitricemia*: No anti-cancer therapy during observation | | Anti-cancer therapy concurrently or within past 6 months | |
|---|---|---|---|---|
| Source of cancer | Total cases | Elevated (*) | Total cases | Elevated (*) |
| Breast | 18 | 9 | 28 | 11 |
| Other genito-urinary | 4 | 3 | 13 | 5 |
| Respiratory | 8 | 7 | 11 | 4 |
| Gastro-intestinal | 6 | 2 | 3 | 2 |
| Mesenchymal | 5 | 2 | 5 | 1 |
| Totals | 41 | 23 (55%) | 60 | 23 (38%) |

* $>$ 2 $\times$ S.D. of normal mean venous citrate level (95 per cent level of confidence).

increase with time, especially during the second six months of prednisone therapy (Table IX). Phosphohexose isomerase analyses showed a similar trend. The data are insufficient to draw conclusions from either alkaline phosphatase or transaminase measurements. Citrate concentration, however, decreased during the first six months of prednisone treatment of breast cancer, to values well within the normal range, before a later secondary rise, suggesting different factors influenced venous concentration of the latter during treatment compared to various enzymatic functions. Other types of metastatic cancer in men and women also often had abnormal acid phosphatase and phosphohexose iseromase activity as shown by the mean values, although the variation of activity from case to

TABLE IX.—*Enzyme Activity of Venous Blood and Citrate Concentration*

| Group | Sex | Alkaline phosphatase (Bodansky units) | Copper resistant acid phosphatase (μmole phenol/ 100 ml.) | Phosphohexose isomerase (Bodansky units) | Glutamic oxalacetic transaminase (units) | Fasting citrate concentration (μg./ml.) |
|---|---|---|---|---|---|---|
| Normal . . . | M. | Under 5 | Under 24 μmole/ 100 ml. | Under 40 | Under 20 units | 27·2±1·3 (27) |
| | F. | .. | .. | .. | .. | 38·3±1·8 (44) |
| Metastatic carcinoma of breast No therapy | ,, | 5·95±1·13 S.E. (19) | 38·8±4·2 S.E. (27) | 42·6 (22) | 17·5 (8) | 45·2±3·8 S.E. (29) |
| Same, prednisone therapy 6 months or less | | 7·3 (3) | 40·1±6·9 (13) | 69·2±18·6 (10) | 15 (4) | 33·6±2·9 (13) |
| Same, prednisone for over 6 months | | 14·6 (5) | 46·8±4·9 (10) | 63·3±52 (9) | 35·8 (4) | 44·3±5·9 (12) |
| Other metastatic carcinoma No therapy | M. | 5·8±1·7 (12) | 33·3±5·6 (19) | 41·6±5·6 (14) | 12 (3) | 43·9±3·8 (25) |
| Other metastic carcinoma No therapy | F. | 8·2±3·2 (6) | 32·8±4·6 (10) | 53·4±14·5 (10) | 12·7 (3) | 39·9±5·0 (15) |

Numbers in parentheses indicate number of patients tested, upon which mean ± S.E. is based.

case was great. In this latter group, enzymatic abnormalities also appeared to vary independently of citrate concentration in many individual cases.

10. *Reduction of elevated citrate concentration in mammary cancer by anti-estrogenic therapy with prednisone*

During these studies, hormonal therapy consisting of oophorectomy in patients under 64 years of age, combined with pituitary-adrenocortical suppression utilizing prednisone 20–30 mg. daily was given to many of the new advanced breast cancer cases (Lemon, 1959). Serial observations of the immediate effects of prednisone administration upon citrate dynamics were made in 7 healthy volunteers and in 10 cancer patients. An inconsistent change in fasting venous citrate was noted in healthy volunteers receiving prednisone, while the cancer patients with hypercitricemia (including one with bronchogenic carcinoma) in nearly all cases had a significant (p = < ·01) reduction of citrate concentration to normal female or even normal male values during the first 6 months of treatment (Table IX). This fall in venous citric acid concentration was accompanied by a mean 28 per cent reduction in 18 estimations of the clearance rate of injected citric acid in chiefly female cancer patients, whose gonads had been ablated (Tables III, X). The less regular reduction of citrate clearance rate was noted in healthy volunteers, whose intact gonads possibly partially nullified the anti-estrogenic effects of prednisone therapy, and whose citrate concentrations were initially within the normal range. These observations appear consistent with a cortisone inhibition of citrate diffusion into blood in cancer patients, leading to a reduction of blood citrate concentration in spite of reduced citrate uptake by tissues.

It was striking to note the recurrently elevated blood citrate values when patients relapsed while on adrenal corticoid therapy (Table XI).

TABLE X.—*Effect of Prednisone Therapy on Citrate Clearance (C) in Volunteers and Cancer Patients*

| Healthy volunteers (Male) | Duration therapy | Per cent change from pre-therapy observation | Cancer patients (Male) | Origin | Duration therapy | Per cent change from pre-therapy observation |
|---|---|---|---|---|---|---|
| J. L— | 3 days | +55 | F. C— | Lung | 8 days | −70 |
| J. S— | ,, | +75 | | | | |
| B. I— | ,, | −12 | | | | |
| R. N— | ,, | −37 | (Female) | | | |
| | | | E. W— | Breast | 1 day | +31 |
| (Female) | | | I. G— | Endometrium | 27 days | −63 |
| L. K— | ,, | —11 | H. A— | Breast | 2 days | −13 |
| R. D— | ,, | —36 | ,, | ,, | 6 weeks | +22 |
| V. D— | ,, | −62 | ,, | ,, | 2½ months | −41 |
| | | | P. I— | ,, | 6 days | −61 |
| | | | ,, | ,, | 2 months | −43 |
| | | | ,, | ,, | 5 months | +18 |
| | | | ,, | ,, | 6 months | +31 |
| (Dosage | | | B. L— | ,, | 9 days | −46 |
| =30 mg./day) | | | B. T— | ,, | 11 days | −67 |
| | | | L. M— | ,, | 9 months | + 3 |
| | | | ,, | ,, | 1 year | −24 |
| | | | A. M— | ,, | 12 days | −14 |
| | | | M. C— | ,, | 6 months | −90 |
| | | | M. L— | ,, | 9 days | —60 |
| | | | ,, | ,, | 2 weeks | −22 |
| 7 patients | | Mean = −4% (7 tests) | 11 patients | | | Mean = −28% (18 tests) |

TABLE XI.—*Recurrence of Hypercitricemia During Clinical Relapse of Advanced Mammary Cancer Treated by Prednisone*

| Patient | Metastases | Initial venous citrate (μg./ml.) | Mean, range of venous citrate duration theraputic remission | Duration therapy | Venous citrate during terminal relapse (therapy continued) (μg./ml.) | Time before death |
|---|---|---|---|---|---|---|
| H. A— | Bone, liver later | 64·8 | 30·5 (23·1–37·2) | 6 months | 62·3 | 1 month |
| B. L— | Bone, lung | 64·8 | 49·4 (47·4–51·4) | 1 month | 103·0 | 2 weeks |
| H. C— | Brain, lung | 100+ | 20·2 (18·5–21·9) | 2 months | 100+ | 1 month |
| K. L— | Lung, mediastinum | 69·5 | 50·0 | 2 months | 30·4 | 1 month |
| L. M— | Skin, bone, | 50 | 34·8 (27·4–41·9)* | 1 year | 76·1* | 2 months |
| | liver later | . . | 34·1 (30·2–38·0) | . . | 116·6 | 1 week |
| L. T— | Bone, lung | 79·0 | 30·1 (18·8–40·3) | 18 months | 23·9 | 1 month |
| P. I— | Bone | 57·2 | 29·4 (19·4–39·1) | 7 months | 75·9 (62–95·5) | 3 months |
| F. C— | ,, | 89·5 | 34·5 (22·2–49·7) | 18 months | 47·9 (26·9–64) | 7 months |
| M. L— | ,, | 100+ | 39·4 (21·6–60·8)* | 2 years | 79·2 (66–106)* | . . |
| | | | 31·5 (16·3–39·1) | . . | 39·1 | (Living) |
| Mean of patients means : | | 75 | 35·0 | . . | 65·9 | . . |

* Temporary relapse.

### DISCUSSION

Hypercitricemia in various types of cancer has not been frequently noted by several previous observers (Schersten, 1931 ; Rottino, Hoffman and Brondolo, 1952) with a single undocumented exception (Kyle and Canary, 1957). Review of the case material utilized in several of these papers indicates that only a small number of patients were studied, of whom many had already received anti-cancer therapy. Furthermore, it is not clear whether fasting bloods were always utilized for analyses, which will usually yield the peak venous citrate concentrations. Relatively few case reports are included of breast, prostate and lung carcinomata which comprise the majority of our patients with hypercitricemia. Simultaneous pre-selected control groups were not used in some previous studies, retrospective controls being used. These differences appear to explain most of the discrepancies between our observations, and previous reports.

In spite of inflammatory disease involving bone and joints in rheumatoid arthritis and metabolic disorders, such as post-menopause osteoporosis, serum citrate was normal in all non-cancer cases. Atrophic bone changes were frequently widespread as shown by X-ray at the time of our sampling. This suggests that citrate measurement can be a useful adjunct in differential diagnosis of the benign or malignant nature of some osseous lesions, in which demineralization is a prominent feature.

From our observations hypercitricemia appears to be a relatively common disorder usually independent of hypercalcemia in patients with advancing neoplastic disease invading liver, lung or bone among other tissues. Hypercitricemia during active cancer growth is of particular interest in that it may represent a dysfunction of the Krebs cycle through excessive citrate production or deficient citrate utilization via condensation to acetoacetate, either in cancer or normal tissues or both. Destruction of the activity of Coenzyme II (TPN) might result in such a disturbance. In addition citrate equilibrium in osseous tissues may be disturbed, as seen in hyperparathyroidism or Paget's disease with hypercitricemia secondary to osteolysis (Kissin and Kreeger, 1954 ; Chang and Freeman, 1950*b*). In this latter case, a negative calcium balance and hypercalcemia might be expected to accompany hypercitricemia, as we have observed in one case of functioning parathyroid carcinoma and in occasional mammary cancer patients. Intensive osseous destruction, although present roentgenologically in many of the hypercitricemia breast cancer patients, was not severe enough to induce hypercalcemia in most of these patients and may be coincidental, rather than contributory.

Gomori and Gulyas (1944) were the first to observe that parenteral administration of sodium citrate in dogs leads to marked hypercalcuria with minimal alteration in serum calcium concentration. Their work has been confirmed (Chang and Freeman, 1950*a*), indicating that in the absence of hyperparathyroidism and its attendant metabolic disturbance hypercitricemia may produce a ten to twenty fold augmentation of calcium excretion without raising total serum calcium concentration above 12 mg. per cent. This may be the result of a marked increase in the ultra-filterable fraction of plasma calcium which is citrate bound, or because of diminished tubular reabsorption of the calcium citrate complex. These observations however help to explain the rare coexistance of hypercitricemia and hypercalcemia in our cancer population.

Hormonal factors such as insulin (Pincus, Natelson and Lugovoy, 1949), epinephrine (Pincus, Natelson and Lugovoy, 1951) and adrenocortical hormones (Pincus, Natelson and Lugovoy, 1951 ; Agrell, Lindell and Westling, 1955) also have been shown to alter venous citrate concentration. None of our hypercitricemic cancer patients was diabetic or receiving insulin, nor did we encounter any pheochromocytomas in our series of cases. Although adrenocortical insufficiency results in hypercitricemia in man (Martenesson, 1949), none of our patients was suffering from acute adrenocortical insufficiency at the time of our studies, and only one patient with leiomyosarcoma metastatic to the adrenal glands was receiving adrenal steroid therapy, as a result of a previous Addisonian crisis. At the time of our study this patient was in electrolyte balance. Although the kidneys serve as a major site of citrate uptake from blood, significant impairment of renal function was not present in our hypercitricemia patients. Hepatic insufficiency was also absent in most hypercitricemia patients, including those with hepatic metastases. No evidence was obtained that hypercitricemia was related to any reduction of citrate uptake fiom blood (Table III).

The striking reduction of venous citrate concentrations to normal in previously hypercitricemia breast cancer patients receiving cortisone or prednisone, occurred in spite of a 28 per cent reduction in clearance rate for injected citrate. This depression of citrate uptake from blood by adrenal steroid therapy in all likelihood is closely related to the reduction of acetate utilization which has also been reported. Hennes and Shreeve administered doses of prednisone identical to those we have utilized, to patients receiving $^{14}$C-labelled acetate, and noted an initial reduction of 20–30 per cent in rate of radiocarbon excretion compared to control observations (Hennes and Shreeve, 1959). Over a 24 hour period a 10–15 per cent reduction of cumulative radioactivity excretion was demonstrated. Henneman and Bunker have reported elevated venous lactate and pyruvate concentrations following adrenal steroid therapy and in Cushings' syndrome (Henneman and Bunker, 1957), suggesting a decrease in pyruvate oxidation under these circumstances. Impairment of glucose tolerance has long been recognized as one of the most dependable laboratory manifestations of adrenal cortical hyperfunction.

The control of hypercitricemia by adrenal steroid therapy must be accounted for by reduced citrate diffusion into blood from some tissue source. Not only must this diffusion rate be very high prior to therapy to induce hypercitricemia, in view of the 20–25 g./day capacity of the body to utilize citrate, but steroid induced inhibition of diffusion from this tissue source must far exceed the net overall reduction in uptake of citrate caused by prednisone therapy. Our observations indicate that a major source of citrate enrichment of blood exists in the sinusoids of bone marrow, where at all times citrate concentration exceeds that of mixed peripheral arterial or venous blood. Adrenal steroid therapy appears to have a more marked and consistent effect reducing venous citrate of breast cancer patients than that of healthy volunteers. However, differences in the duration and intensity of therapy or in the degree of sex hormone inhibition so induced, may account for this variation in response. The greater prevalence of hypercitricemia in advanced breast, prostate and lung carcinoma patients in whom osseous metastases are so common (Table III) and the infrequency of occurrence in benign tumors or localized breast cancer suggest that hypercitricemia is potentiated by widespread neoplastic invasion of bone marrow. The simultaneous elevation of venous acid phosphatase in hypercitricemic patients also supports bone marrow invasion as the chief source for excessive diffusion of citrate into blood, since this enzyme is elevated in venous blood in 75 per cent of patients with osseous metastases of breast or prostate carcinoma prior to therapy (Reynolds, Lemon and Byrnes, 1956). We have also found that marrow sinusoid blood is far higher in acid phosphatase activity, than peripheral arterial or venous blood (Reynolds, Lemon, Kaplan, Idelson, Mueller and Derow, 1959). Likewise, abnormal venous phosphohexose isomerase activity is often present in mammary carcinoma with osseous invasion (Bodansky, 1954*a*), and elevated alkaline phosphatase activity is well known to result from osseous or hepatic invasion by tumour.

Since the early reports of Dickens and others concerning the presence of citrate in the organic matrix of vertebrate bone (Dickens, 1941 ; Thunberg, 1953), which contains 95 per cent of total body citrate, a great deal of work has been carried out showing a close relationship between calcium and citrate metabolism in response to various stimuli, and a current hypothesis of bone formation includes precipitation of calcium citrate on the superficial lamellae of bone trabeculae (Neumann and Neumann, 1958). Enzymes necessary for local production of citrate have been described in osteoid tissue (Dixon and Perkins, 1952). One observed case of osteogenesis imperfecta, in a 5 year old boy, had a venous citrate of 89·5 $\mu$g./ml. prior to therapy, falling to a normal value after testosterone therapy had induced some calcification and clinical improvement. The movement of calcium and citrate in and out of bone under the influence of parathormone or Vitamin D therapy is generally in the same direction (Carlson and Hollunger, 1954 ; Elliott and Freeman, 1956), and a similar trend is apparent in our data. With the exception of prostatic carcinoma which rarely induces hypercalcemia or hypercalcuria, cancer frequently resulting in hypercitricemia such as lung or breast are also prone to develop hypercalcemia. When the latter develops, hypercitricemia usually co-exists. The " Idiopathic " hypercalcemia reported in advanced lung, breast, ovarian or renal cancer cases in the absence of detectable osseous metastases may be possibly associated with hypercitricemia, if the latter abnormality were to be looked for (Plimpton and Gellhorn, 1956). A calcium-binding substance has been postulated in those cases in whom an elevated serum alkaline phosphatase suggested bone disease.

Normal prostatic and mammary epithelium secrete extremely high concentrations of citric acid into their respective secretions as a result of specific sex hormonal stimulation (Mann, 1954 ; Lenner, 1934) and this function possibly is preserved in some endocrine dependent cancers. Talalay and Williams-Ashman have postulated that estrogenic cellular stimulation is mediated via effects upon the balance of pyridine nucleotide dependent transdehydrogenase systems involved in the function of the Krebs cycle (Talalay and Williams-Ashman, 1958), which would therefore govern the production and/or utilization of citrate by hormone sensitive tissues.

Our one negative observation of citrate diffusion from breast cancer occurred in a patient with primary disease lacking demonstrable lymphatic or osseous metastases and whose venous blood levels were normal. If tumor cells in bone marrow served as a major source for the high citrate concentrations we have noted, osteolytic bone destruction might be explained by the reversal of the normal processes of calcification, through creation of excessive amounts of free citrate in the diseased sinusoids to compete with phosphate for the calcium hydroxyapatite (Neumann and Neumann, 1958). Neoplastic invasion of soft tissues may also be facilitated by citrate induced solvation of procollagen (Jackson, 1957).

Numerous observations attest to the striking ability of cortisone and prednisone therapy to control hypercalcemia in cancer, and to induce recalcification of osseous metastases in breast cancer, coincidental with reduction of circulating citrate concentration to normal (Lemon, 1956, 1957, 1959 ; Nissen-Meyer, 1957). Adrenal corticoids have been shown to antagonize the action of estrogens upon hormone-dependent target tissues such as endometrium, nullifying both water inhibition and growth (Szego and Roberts, 1953 ; Huggins and Jenson, 1955 ; Velardo, Hisaw and Bever, 1956) as well as exerting a depressing influence upon the growth of a number of different types of transplantable and spontaneous cancers (Pearson, Li, MacLean, Lipsett, and West, 1955 ; Rusch, 1956). As a working hypothesis it may be postulated that the normalization of venous citrate and subsequent recalcification of some osseous metastases may also represent the result of direct inhibitory effects exerted upon osseous mammary carcinoma metastases as well as indirect effects secondary to depressed sex hormone secretion. Our data is insufficient as yet concerning the influence of steroid therapy upon hypercitricemia in other types of metastatic cancer to draw further inferences. Although clinically obvious metastases were noted in hepatic or pulmonary or other areas without radiologically detectable bone involvement in 71 per cent of our hypercitricemia cases with metastatic cancer (Table VII) the prevalence of circulating tumour emboli in over 50 per cent of patients with malignant neoplasms insures that the sinusoids of bone marrow become the repository of active or inactive metastases, sooner or later in most forms of advanced cancer (Fisher and Turnbull, 1955 ; Moore, Sandberg and Schubarg, 1957 ; Engell, 1955).

Finally, hypercitricemia to the levels which we have observed must produce some major disturbances of membrane permeability, resulting from alteration of the mono-valent to di-valent cation ratio through calcium binding by elevated citrate. Normally female venous blood contains about 0·6 m-equiv./l. of citrate (38 $\mu$g./ml.) capable of binding a similar amount of ionized calcium (Hastings, MacLean, Eichelberger, Hall and Da Costa, 1934). About 65 per cent of serum calcium is normally ultra-filterable or 3·24 m-equiv./l. (Neumann and Neumann, 1958). Bicarbonate, phosphate and citrate are the principal anions available to bind diffusible calcium. Under normal circumstances, ionized calcium averages about 2·66 m-equiv./l., so that citrate bound serum calcium constitutes a labile fraction amounting to nearly one-sixth of total diffusible calcium. If citrate concentration rises to the values herein reported, then 1·0–1·8 m-equiv./l. or more of serum calcium ion might be complexed with citrate, assuming no shift of the latter from protein, leaving only an estimated 1–2 m-equiv./l. of ionized calcium for control of membrane permeability. Hastings and co-workers clearly showed that calcium citrate was metabolically inert in so far as the frog heart was concerned, up to values as high as 20 m-mole/l. (Hastings, MacLean, Eichelberger, Hall and Da Costa, 1934), and hence cannot function in the control of membrane permeability.

Contrary to some earlier reports (Allen, Clark, Thornton and Adams, 1944) hypercitricemia has been observed as a hazardous complication of exchange transfusion in infants (Wexler, Pincus, Natelson and Lugovoy, 1949), and of massive blood replacement therapy in adults with hepatic cirrhosis and bleeding varices, major vascular surgery and major operations in the portal area (Bunker, Stetson, Coe, Grillo and Murphy, 1955). Bunker and co-workers observed that the immediate rise in serum citrate after infusion of varying amounts of citrated blood varied with the rate of administration and total amount of blood infused. Somewhat higher arterial blood levels were observed in patients with hepatic disease, than in other pre-operative patients under pre-medication. Values were noted as high as 300–400 $\mu$g./ml. in their series ($\pm$ 5·0–6·5 m-equiv./l.). Depression of ionic calcium concentration calculated on the basis of total calcium, total protein and citrate concentration (Hastings, MacLean, Eichelberger, Hall and Da Costa, 1934) to values as low as 1·10–1·40 m-equiv./l. was associated with hypotension or shock in the majority of cases, a few of whom responded to intravenous calcium ion therapy. Only rarely did their patients show tetanic manifestations which we have not observed as yet, either. With calculated ionic calcium above 1·50 m-equiv./l., no hypotensive phenomena were observed. Less deleterious subjective effects may result from smaller elevations of serum citrate such as we have noted, which consisted primarily of anorexia and nausea, and emphasize that hypercitricemia may contribute to malaise of the cancerous patient. Reduction of blood citrate levels to normal by prednisone therapy, and the simultaneous striking subjective improvement of many patients, including their strength and appetite, may be related to a restoration of a more normal ionic cellular environment.

In most of the adult surgical patients in whom massive replacement of blood is necessary, extreme activation of the pituitary-adrenocortical system by the underlying catastrophic disease probably contributed to hypercitricemia, by impairing the metabolism of exogenous citrate, such as we have observed occurs after adrenal cortical steroid therapy.

Cookson and co-workers have suggested that hypothermia which has been utilized in vascular surgery may compound the hazard by further reduction of ability to utilize large amounts of administered citrate (Cookson, Costas-Durieux and Bailey, 1954). Anoxia *per se* may also induce hypercitricemia (Hallman and Forsander, 1952).

Bunker and co-workers obtained fairly satisfactory agreement between the observed increment in arterial citrate concentration after infusion (average in 11 patients=13·7 mg./100 c.c.) and the increment predicted on the basis of equilibration in extracellular fluid within 6–16 minutes (average=11·2 mg./100 c.c.) If the latter figure is corrected for the mean rate of fall we have noted in serum citrate concentration after termination of infusion, approximately 1 $\mu$g./ml./minute in both sexes in the period 5–30 minutes after injection, the expected citrate concentration would be 12·5 mg./100 c.c. from their data, on the assumption that the clearance process begins immediately with initial elevation of serum citrate. It is likely that the amount of protein bound calcium was overestimated from the total protein concentrations observed in many of the patients with hepatic cirrhosis in the series reported by Bunker *et al.* (1955) in whom a considerable increase in gamma globulin fraction could be expected, which has been found to have only slight calcium binding capacity (Carr, 1955). Therefore, their estimates of residual ionic calcium may be somewhat on the low side. Not all recent investigators have found hypercitricemia after massive transfusion, no doubt due partly to the very rapid utilization of citrate which all have noted, even with advanced liver disease (Howland, Bellville, Zucker, Boyan and Cliffton, 1957).

### SUMMARY

Citric acid concentration has been measured using the pentobromoacetone procedure in blood from bone marrow, arterial and venous sites, in 358 healthy volunteers, arthritics and patients with various types of cancer in all stages. The clearance of exogenous citrate from blood has been estimated in a representative fraction of each group of patients and approximates 0·9 g. per hour. Women have been found to have consistently higher fasting venous serum citric acid than men, with a slightly lower citrate clearance rate. Bone marrow blood consistently shows the highest concentration of citrate, followed by arterial and finally venous blood. Venous citric acid concentrations greater than twice the S.D. from the mean for each sex (" hypercitricemia ") occurred in 4·2 per cent of healthy volunteers, in 4·6 per cent of all patients with benign diseases, in 11·1 per cent of patients with pre-malignant tumors, and in 23·6 per cent of patients with cancer. Anorexia and weakness were generally noted in the latter group. Hypercalcemia exceeding 12 mg. per cent (6 m-equiv./l.) was noted in only 3 patients in the entire series, but abnormal acid phosphatase and lactic dehydrogenase activity usually accompanied hypercitricemia. Advanced carcinomas of the breast, prostate, and lung were most commonly associated with hypercitricemia, probably as a result of osseous metastases. Prednisone therapy in advanced mammary carcinoma usually reduced elevated blood citric acid to normal simultaneously reducing by 28 per cent the utilization of administered citrate, indicating the probable suppression of tumor growth in osseous and perhaps in other metastatic sites.

These studies have been supported by a research grant from the National Cancer Institute, by the Rebecca Rice Memorial Grant of the American Cancer Society to Boston University, by a grant from the Quincy United Fund, of Quincy, Massachusetts, and a student research fellowship from the Massachusetts Arthritis and Rheumatism Foundation.

### REFERENCES

Agrell, I., Lindell, S. E. and Westling, H.—(1955) *Acta physiol. scand.*, **34**, 135.

Allen, J. G., Clark, D. E., Thornton, T. F. and Adams, W. E.—(1944) *Surgery*, **15**, 824.

Bodansky, A.—(1932) *J. biol. Chem.*, **99**, 197

Bodansky, O.—(1954*a*) *Cancer*, **7**, 1191.—(1954*b*) *Ibid.*, **7**, 1200.

Bunker, J. P., Stetson, J. B., Coe, R. C., Grillo, H. C. and Murphy, H. D.—(1955) *J. Amer. med. Ass.*, **157**, 1361.

Carlson, A. and Hollunger, G.—(1954) *Acta physiol. scand.*, **31**, 317.

Carr, C. W.—(1955) in ' Electrochemistry in Biology and Medicine ', Shedlovsky, T. (ed.). Chapter 14. New York (John Wiley and Sons).

Chang, T. S. and Freeman, S.—(1950*a*) *Amer. J. Physiol.*, **160**, 330.—(1950*b*) *Ibid.*, **160**, 341.

Clark, E. P. and Collip, J. B.—(1925) *J. biol. Chem.*, **63**, 461.

Cookson, B. A., Costas-Durieux, J. and Bailey, C. P.—(1954) *Ann. Surg.*, **139**, 430.

Cori, C. F. and Cori, G. T.—(1925) *J. biol. Chem.*, **65**, 397.

DICKENS, F.—(1941) *Biochem. J.*, **35**, 1101.
DIETRICH, L. A. AND SHAPIRO, D. M.—(1956) *Cancer Res.*, **16**, 585.
DIXON, T. F. AND PERKINS, H. R.—(1952) *Biochem. J.*, **52**, 260.
ELLIOTT, J. R. AND FREEMAN, S.—(1956) *Endocrinology*, **59**, 200.
ENGELL, H. C.—(1955) *Acta chir. scand.*, Supp. 201, 1.
ETTINGER, R. H., GOLDBAUM, L. R., SMITH, L. H.—(1952) *J. biol. Chem.*, **199**, 531.
FISHER, E. R. AND TURNBULL, R. B.—(1955) *Surg. Gynec. Obstet.*, **100**, 102.
FISKE, C. H. AND SUBBAROW, Y.—(1925) *J. biol. Chem.*, **66**, 375.
FRANCO.—(1957) ' Sigmo-Franco Procedure ', *Sigma Chem. Co. Tech. Bulletin*, No. 505.
GOMORI, G. AND GULYAS, E.—(1944) *Proc. Soc. exp. Biol. N.Y.*, **56**, 226.
HASTINGS, A. B., MACLEAN, F. C., EICHELBERGER, L., HALL, J. L. AND DA COSTA, E. —(1934) *J. biol. Chem.*, **107**, 351.
HALLMAN, N. AND FORSANDER, O.—(1952) *Ann. Med. exp. Fenn.*, **30**, 287.
HENNEMAN, D. H. AND BUNKER, J. P.—(1957) *Amer. J. Med.*, **23**, 34.
HENNES, A. R. AND SHREEVE, W. W.—(1959) *Proc. Soc. exp. Biol. N.Y.*, **100**, 246.
HERNDON, R. F. AND FREEMAN, S.—(1958) *Amer. J. Physiol.*, **192**, 369.
HILL, B. R. AND LEVI, C.—(1954) *Cancer Res.*, **14**, 513.
HOWLAND, W. S., BELLVILLE, J. W., ZUCKER, M. B., BOYAN, P. AND CLIFFTON, E.—(1957) *Surg. Gynec. Obstet.*, **105**, 529.
HUGGINS, C. AND JENSON, E. V.—(1955) *J. exp. Med.*, **102**, 347.
JACKSON, D. S.—(1957) ' Connective Tissue. A Symposium '. Tunbridge *et al.* (ed.). Oxford (Blackwell Scientific Publications), p. 62.
KISSIN, B. AND KREEGER, N.—(1954) *Amer. J. med. Sci.*, **228**, 301.
KYLE, L. H. AND CANARY, J. J.—(1957) *J. Lab. clin. Med.*, **49**, 590.
LEMON, H. M.—(1956) *Clin. Congr. Amer. Coll. Surg.*, **6**, 415.—(1957) *Annals intern. Med.*, **46**, 457.—(1959) *Cancer*, **12**, 93.
*Idem*, DAVISON, M. D. AND ASIMOV, I.—(1954) *Ibid.*, **7**, 92.
*Idem*, MUELLER, J. H,, LOONEY, J. M. CHASEN, W. H. AND KELMAN, M.—(1959) *Boston med. quart.*, **10**, 1.
LENNER, A.—(1934) *Acta obstet. gynec. scand.*, *Supp.* 1 ; **14**, 1.
MANN, T.—(1954) ' The Biochemistry of Semen '. London (Methuen and Co., Ltd.), p. 16.
MARKS, P. A. AND BISHOP, J. S.—(1957) *J. clin. Invest.*, **36**, 254.
MARTENSSON, J.—(1949) *Acta med. scand.*, **134**, 61.
MILLER, H. AND CARRUTHERS, C.—(1950) *Cancer Res.*, **10**, 636.
MOORE, G. E., SANDBERG, A. AND SCHUBARG, J. R.—(1957) *Ann. Surg.*, **146**, 580.
NEUMANN, W. F. AND NEUMANN, M. W.—(1958) ' The Chemical Dynamics of Bone Mineral '. (Univ. of Chicago Press), p. 209.
NISSEN-MEYER, R.—(1957) *Acta endocr.*, *Supp.* 31, p. 314.
PEARSON, O. H., LI, M. C., MACLEAN, J. P., LIPSETT, M. B. AND WEST, C. D.—(1955) *Ann. N.Y. Acad. Sci.*, **61**, 393.
PINCUS, J. B., NATELSON, S. AND LUGOVOY, I. K.—(1949) *J. clin. Invest.*, **28**, 741.—(1951) *Proc. Soc. exp. Biol. N.Y.*, **78**, 452.
PLIMPTON, C. H. AND GELLHORN, A.—(1956) *Amer. J. Med.*, **21**, 750.
POTTER, V. R. AND BUSCH, H.—(1950) *Cancer Res.*, **10**, 353.
RECHENBERGER, J. AND BENNDORF, S.—(1956) *Z. Altersforsch.*, **10**, 49.
REYNOLDS, M. D., LEMON, H. M. AND BYRNES, W. W.—(1956) *Cancer Res.*, **16**, 943.
*Idem*, LEMON, H. M., KAPLAN, G. A., IDELSON, B. A., MUELLER, J. AND DEROW, M. A. —(1959) *Proc. Amer. Ass. Cancer Res.*, **3**, 56.
ROTTINO, A., HOFFMAN, G. T. AND BRONDOLO, B.—(1952) *Proc. Soc. exp. Biol. N.Y.*, **80**, 339.
RUSCH, H. P.—(1956) *Cancer Res.*, **16**, Supp. 4, p. 183.
SAFFRON, M. AND DENDSTADT, O. F.—(1948) *J. biol. Chem.*, **175**, 849.
SCHERSTEN, B.—(1931) *Skand. Arch. Physiol.*, **63**, 97.

SCHWARTZ, M. K., GREENBERG, E. AND BODANSKY, O.—(1959) *Proc. Amer. Ass. Cancer Res.*, **3**, 61.
SIBLEY, J. A., FLEISCHER, G. A., HIGGINS, G. M.—(1955) *Cancer Res.*, **15**, 306.
STARK, J. B., GOODBAR, A. E. AND OWENS, H. S.—(1951) *Analyt. Chem.*, **22**, 413.
SZEGO, C. M. AND ROBERTS, S.—(1953) *Recent Progr. Horm. Res.*, **8**, 419.
TALALAY, P. AND WILLIAMS-ASHMAN, H. G.—(1958) *Proc. nat. Acad. Sci., Wash.*, **44**, 15.
THUNBERG, T.—(1953) *Physiol. Rev.*, **33**, 1.
VELARDO, J. T., HISAW, F. L., BEVER, A. T.—(1956) *Endocrinology*, **59**, 165.
WEXLER, I. B., PINCUS, J. B., NATELSON, S. AND LUGOVOY, I. K.—(1949) *J. clin. Invest.*, **28**, 474.
WOLFSON, S. K., SPENCER, J. A., STERKEL, R. L. AND WILLIAM-ASHMAN, H. G.—(1958) *Ann. N.Y. Acad. Sci.*, **75**, 260.
YARBO, C. L.—(1956) *J. Urol.*, **75**, 216.